# Mfn2 deletion in brown adipose tissue protects from insulin resistance and impairs thermogenesis

**DOI:** 10.15252/embr.201643827

**Published:** 2017-05-24

**Authors:** Kiana Mahdaviani, Ilan Y Benador, Shi Su, Raffi A Gharakhanian, Linsey Stiles, Kyle M Trudeau, Maria Cardamone, Violeta Enríquez‐Zarralanga, Eleni Ritou, Tamar Aprahamian, Marcus F Oliveira, Barbara E Corkey, Valentina Perissi, Marc Liesa, Orian S Shirihai

**Affiliations:** ^1^ Obesity Research Center Department of Medicine Boston University School of Medicine Boston MA USA; ^2^ Division of Endocrinology Department of Medicine David Geffen School of Medicine at UCLA Los Angeles CA USA; ^3^ Biochemistry Department Boston University School of Medicine Boston MA USA; ^4^ Renal Section Department of Medicine Boston University School of Medicine Boston MA USA; ^5^ Laboratório de Bioquímica de Resposta ao Estresse Instituto de Bioquímica Médica Leopoldo de Meis Universidade Federal do Rio de Janeiro Rio de Janeiro RJ Brazil; ^6^ Department of Clinical Biochemistry School of Medicine Ben Gurion University Beer‐Sheva Israel

**Keywords:** brown adipose tissue, insulin resistance, Mitofusin 2, obesity, thermogenesis, Membrane & Intracellular Transport, Metabolism

## Abstract

BAT‐controlled thermogenic activity is thought to be required for its capacity to prevent the development of insulin resistance. This hypothesis predicts that mediators of thermogenesis may help prevent diet‐induced insulin resistance. We report that the mitochondrial fusion protein Mitofusin 2 (Mfn2) in BAT is essential for cold‐stimulated thermogenesis, but promotes insulin resistance in obese mice. Mfn2 deletion in mice through Ucp1‐cre (BAT‐Mfn2‐KO) causes BAT lipohypertrophy and cold intolerance. Surprisingly however, deletion of Mfn2 in mice fed a high fat diet (HFD) results in improved insulin sensitivity and resistance to obesity, while impaired cold‐stimulated thermogenesis is maintained. Improvement in insulin sensitivity is associated with a gender‐specific remodeling of BAT mitochondrial function. In females, BAT mitochondria increase their efficiency for ATP‐synthesizing fat oxidation, whereas in BAT from males, complex I‐driven respiration is decreased and glycolytic capacity is increased. Thus, BAT adaptation to obesity is regulated by Mfn2 and with BAT‐Mfn2 absent, BAT contribution to prevention of insulin resistance is independent and inversely correlated to whole‐body cold‐stimulated thermogenesis.

## Introduction

In response to cold exposure, brown adipose tissue (BAT) activates a thermogenic program, mediated by adrenergic signaling, which increases lipolysis and fat oxidation controlled by proton leak through uncoupling protein 1 (Ucp1) 1, 2, 3. The unique capacity of BAT to generate heat from uncoupled fat oxidation led to the proposal, decades ago, that BAT manipulation could be a therapeutic target to treat obesity and type 2 diabetes.

On the other hand, obesity itself profoundly changes BAT thermogenic function in different directions. While previous studies suggested that obesity‐induced lipohypertrophy of BAT is mediated by mitochondrial dysfunction, impaired fat oxidation, and thermogenic capacities in BAT 4, recent findings suggest that BAT lipohypertrophy and reduced thermogenesis may represent an adaptive response that can be beneficial for glucose homeostasis 5.

An unexplored aspect of BAT function is the contribution of mitochondrial dynamics in the response of BAT to obesity. Previous studies demonstrate a tight correlation between changes in mitochondrial morphology and respiratory function 6, 7. In this context, Mitofusin 2 (Mfn2), a protein mediating mitochondrial fusion, is upregulated in BAT after cold exposure or treatment with beta‐3‐adrenergic agonist, showing a positive correlation between BAT thermogenic capacity and Mfn2 8. On the other hand, we have shown that norepinephrine (NE) induces mitochondrial fission in primary brown adipocytes and that the resultant mitochondrial fragmentation is required for proper activation of uncoupled respiration 9. Therefore, mitochondrial fragmentation could either enhance energy expenditure by amplifying Ucp1‐mediated uncoupling and/or be part of Ucp1‐independent thermogenic mechanisms. While the exact mechanism by which Drp1‐mediated fission enhances uncoupling is still elusive, forced fragmentation through short‐term Mfn2 knockdown enhances energy expenditure in brown adipocytes exposed to free fatty acids 9. In the context of this newly identified pathway, we hypothesized that obesity can induce changes in BAT mitochondrial dynamics through Mfn2, thereby affecting energy expenditure and, through that, insulin sensitivity and glucose homeostasis.

To address our hypothesis, we tested the effects of diet‐induced obesity on the mitochondrial fusion protein Mfn2 and on the oxidative function of BAT mitochondria. We find that Mfn2 is increased in BAT from diet‐induced obese mice, along with increased beta‐oxidation capacity. However, Mfn2 activity in BAT contributes to insulin resistance, as Mfn2 excision in brown adipocytes, using Ucp1 promoter‐driven Cre, improves insulin sensitivity in obese mice. Protection from insulin resistance is parallel with gender‐specific changes in BAT function, which promote an enhancement of nutrient utilization capacity supplying ATP in Mfn2‐deleted BAT. This is achieved through increased glycolytic capacity in male BAT and coupled fatty acid oxidation and lipid import capacity in female BAT. These results show that BAT‐Mfn2 loss modulates whole‐body adaptation to obesity, through a competing pathway between insulin sensitivity and thermogenic adaptation to cold. Furthermore, our results show that BAT lipohypertrophy might be an adaptive mechanism to protect from insulin resistance in response to obesity.

## Results

### Diet‐induced obesity increases BAT‐Mfn2 and enhances mitochondrial respiratory capacity of BAT mitochondria

**Figure EV1:**
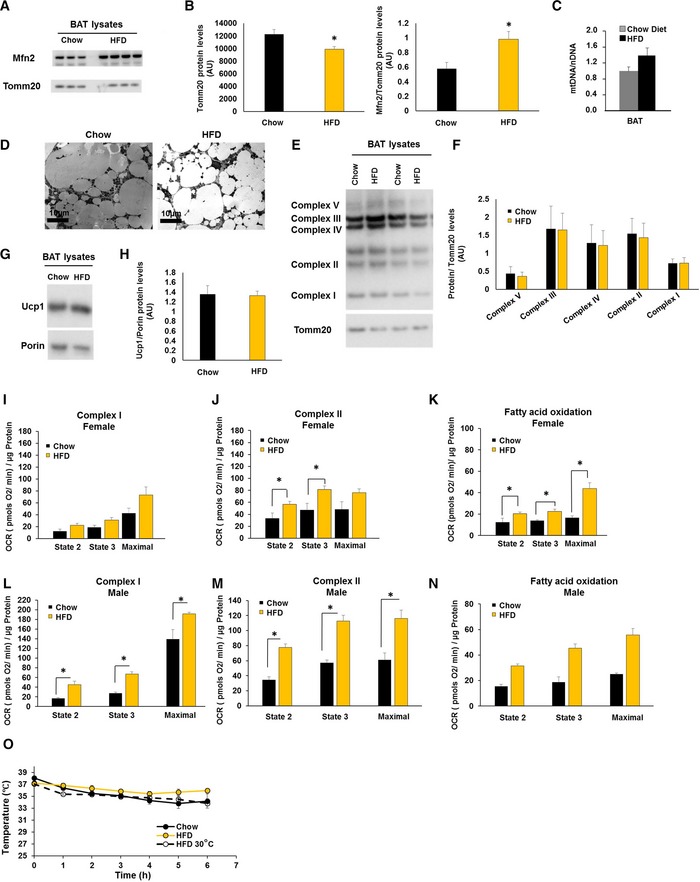
Diet‐induced obesity increases Mfn2 and mitochondrial respiratory capacity in BAT ARepresentative Western blot measuring Mfn2, Tomm20, and Porin on BAT total lysates from wild type female mice fed a chow diet or a high fat diet (HFD).BProtein level quantification of Tomm20 and Porin per microgram of protein loaded, as well as Mfn2 protein levels normalized to their corresponding loading control (Tomm20). Bars represent average of Tomm20, Porin, and Mfn2/Tomm20 from *n* = 3–8 female mice per group ± SEM.CQuantification of mitochondrial DNA (mtDNA) normalized to nuclear DNA (nDNA) by qPCR on extracted DNA from BAT of wild type female mice fed a chow diet or a HFD at 22°C or 30°C. Bars represent average of mDNA/nDNA from *n* = 3 female mice per group ± SEM.DRepresentative EM images of BAT extracted from wild type female mice fed a chow diet or a high fat diet (HFD).ERepresentative Western blot measuring complex I subunit Ndufb8, complex II Sdhb, complex III Uqcrc2, complex IV Cox1, and outer mitochondrial membrane Tomm20 in BAT total lysates from wild type female mice fed a chow or HFD.FProtein level quantification of the four complex subunits normalized by corresponding Tomm20 levels. Bar graphs represent average ± SEM of complexes normalized to Tomm20 from *n* = 5–8 female mice per group fed a chow diet or a HFD.GRepresentative Western blot measuring uncoupling protein 1 (Ucp1) and outer mitochondrial membrane Porin in BAT total lysates from wild type female mice fed a chow or HFD.HProtein level quantification of Ucp1 and the corresponding loading control Porin levels. Bar graphs represent average ± SEM of Ucp1 normalized to Porin from *n* = 5–8 wild type female mice per group fed a chow diet or a HFD.I–KQuantification of oxygen consumption rates (OCR) in BAT isolated mitochondria from wild type female mice fed a chow diet or a HFD under the different respiratory states. State 2 quantifies respiration driven by proton leak (no‐ATP synthesis), state 3 quantifies respiration linked to maximal ATP synthesis, and maximal represents maximal electron transport chain activity induced by FCCP. Bar graphs represent average ± SEM for complex I‐driven respiration (pyruvate + malate, *n* = 4–8 mice per group) (I), complex II‐driven respiration (succinate−rotenone, *n* = 4–8 mice per group) (J), and fatty acid oxidation (palmitoyl carnitine−malate, *n* = 3–6 mice per group) (K).L–NQuantification of OCR in BAT isolated mitochondria from wild type male mice fed a chow diet or a HFD under the different respiratory states. State 2 quantifies respiration driven by proton leak (no‐ATP synthesis), state 3 quantifies respiration linked to maximal ATP synthesis, and maximal represents maximal electron transport chain activity induced by FCCP. Bar graphs represent average ± SEM for complex I‐driven respiration (pyruvate + malate, *n* = 3–13 mice per group) (L), complex II‐driven respiration (succinate−rotenone, *n* = 3–13 mice per group) (M), and fatty acid oxidation (palmitoyl carnitine‐malate, *n* = 2 mice per group).OMouse body temperature measurements from *n* = 5–11 wild type female mice per group at 9 months old and fed a chow diet, a high fat diet (HFD) either at 22°C or at thermoneutrality 30°C. Values shown are means ± SEM.Data information: Statistical analysis: * represents significance using Student's *t*‐test, unpaired *P* < 0.05.

High fat diet (HFD) feeding can activate an adrenergic response increasing Ucp1 and the capacity of BAT to oxidize fat 10. We have previously described that Mfn2 downregulation in primary brown adipocytes from lean mice was sufficient to increase mitochondrial respiration in response to free fatty acids, independently of adrenergic signaling 9. This led us to test the effects of diet‐induced obesity on Mfn2 expression and specifically on BAT mitochondria oxidative capacity. HFD feeding for 40 weeks increased BAT‐Mfn2 protein, without large changes in mitochondrial Tomm20 levels (15% decrease) (Fig EV1A and B). Mitochondrial DNA levels and electron microscopy confirmed absence of large changes in mitochondrial mass induced by HFD in BAT (Fig EV1C and D). No changes were detected in total protein levels of Ucp1 or different subunits from complexes I–IV (Fig EV1E–H).

To address whether HFD translated into functional respiratory changes, we isolated BAT mitochondria and measured respiration driven by different fuels. HFD doubled ATP‐synthesizing (state 3) and maximal respiratory capacity using fatty acids (Fig EV1K and N). We found similar increases with succinate + rotenone (Fig EV1J and M) and pyruvate + malate (Fig EV1I and L). Increased oxidative capacity of BAT mitochondria induced by HFD was parallel to a slight improvement in body temperature maintenance after acute cold exposure (Fig EV1O).

### Deletion of Mfn2 in BAT induces cold intolerance and BAT lipohypertrophy

**Figure 1:**
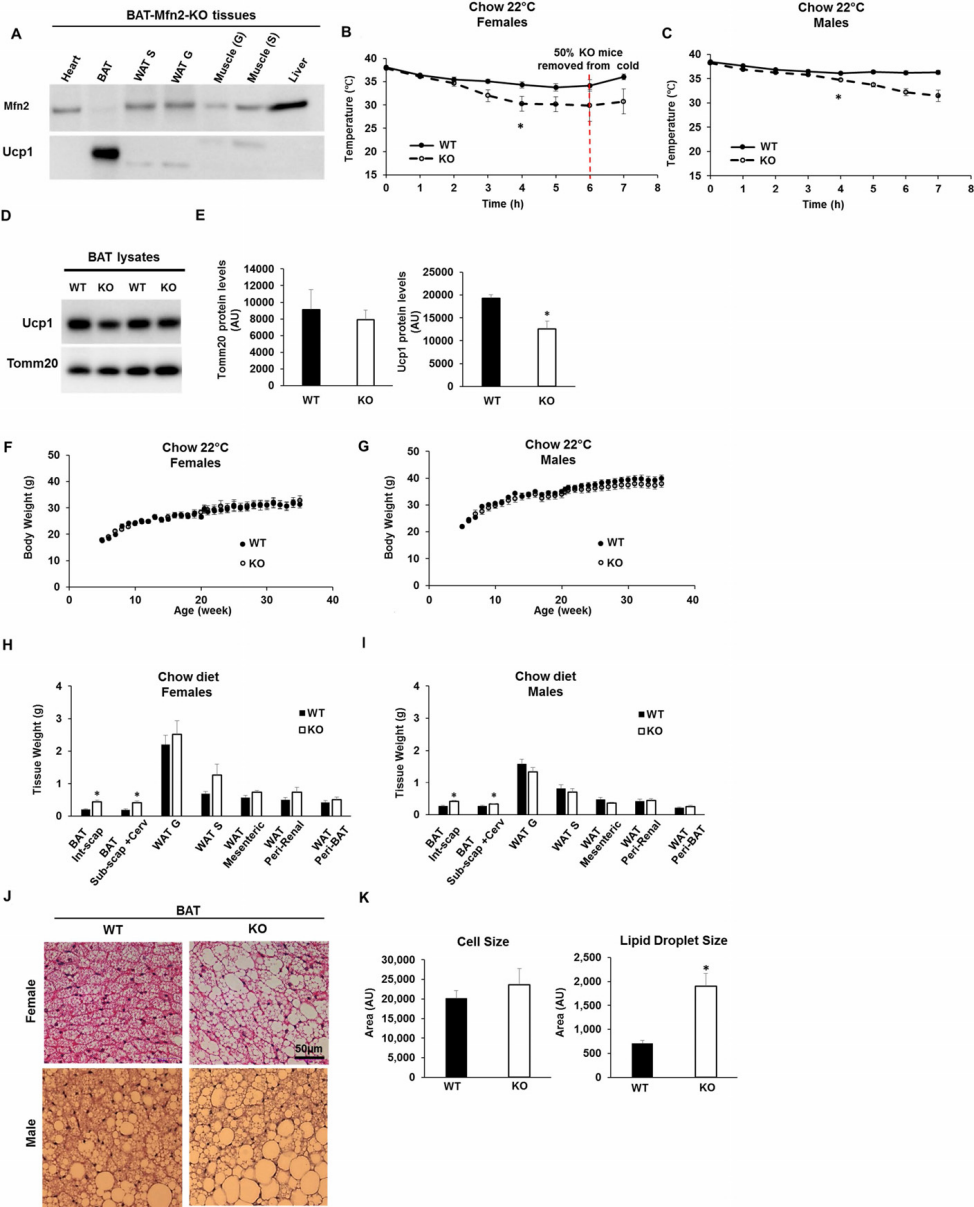
Mfn2 deletion in BAT results in cold intolerance and BAT lipohypertrophy Representative Western blot measuring Mfn2 and Ucp1 in total lysates from different tissues of BAT‐Mfn2‐KO (KO) male mice. WAT S, subcutaneous white adipose tissue; WAT G, perigonadal white adipose tissue. Soleus muscle (S), Gastrocnemius muscle (G).Body temperature measurements before and during cold exposure (4°C) of *n* = 7–9 control and BAT‐Mfn2‐KO female mice per group at 9 months old and fed a chow diet. Values shown are means ± SEM. * represents significance using two‐way ANOVA test WT vs. KO, *P* < 0.05.Body temperature measurements before and during cold exposure (4°C) of *n* = 13–19 control and BAT‐Mfn2‐KO male mice per group at 9 months old and fed a chow diet. Values shown are means ± SEM. * represents significance using two‐way ANOVA test WT vs. KO, *P* < 0.05.Representative Western blot measuring Ucp1 and Tomm20 (mitochondrial loading control) in BAT total lysates from control (WT) and BAT‐Mfn2‐KO (KO) female mice.Protein level quantification of Tomm20 and Ucp1 levels per microgram of protein loaded. Bars represent average of Tomm20 and Ucp1 levels from *n* = 4–5 mice per group ± SEM. * represents significance using Student's *t*‐test, unpaired *P* < 0.05.Body weight measurements of *n* = 7–9 control and BAT‐Mfn2‐KO female mice per group under chow diet over 38 weeks. Values shown are average ± SEM. Two‐way ANOVA test, *P* > 0.05.Body weight measurements of *n* = 13–19 control and BAT‐Mfn2‐KO male mice per group under chow diet over 38 weeks. Values shown are average ± SEM. Two‐way ANOVA test, *P* > 0.05.Quantification of the various WAT and BAT depot weights of *n* = 4–6 control (WT) and BAT‐Mfn2‐KO (KO) female mice (14–15 months old) per group on chow diet. Bar graphs represent average ± SEM. * represents significance using Student's *t*‐test, unpaired, *P* < 0.05.Quantification of the various WAT and BAT depot weights of *n* = 13–19 control (WT) and BAT‐Mfn2‐KO (KO) male mice (14–15 months old) per group on chow diet. Bar graphs represent average ± SEM. * represents significance using Student's *t*‐test, unpaired, *P* < 0.05.Representative images of H&E staining of the BAT sections isolated from control (WT) and BAT‐Mfn2‐KO (KO) female and male mice.Quantification of the brown adipocyte cell size (*n* = 15–25 cells) and lipid droplet size (*n* = 124–250 lipid droplets) from the BAT isolated from *n* = 3–5 control (WT) and BAT‐Mfn2‐KO (KO) male mice per group. Values shown are average ± SEM and are expressed as arbitrary units. * represents significance using Student's *t*‐test, unpaired *P* < 0.05.

To determine the contribution of diet‐induced upregulation of Mfn2 to obesity and glucose intolerance, we deleted Mfn2 specifically in brown adipocytes by generating a BAT‐specific Mfn2 KO. We crossed Ucp1‐cre^+/−^ transgenic mice with Mfn2^flox/flox^ mice to generate Ucp1‐cre^−/−^‐Mfn2^flox/flox^ (control, wild‐type group) and Ucp1‐cre^+/−^‐Mfn2^flox/flox^ mice (BAT‐Mfn2‐KO). Western blot analyses of Mfn2 expression confirmed specific Mfn2 deletion in BAT, maintaining the normal differential distribution of Mfn2 protein levels in other tissues (Fig 1A).

To determine the role of BAT‐Mfn2 in the thermogenic response to cold, body temperature was measured in BAT‐Mfn2‐KO mice housed at 22°C and during acute cold exposure (4°C). Baseline body temperature measured by implanted transponders at 22°C did not show significant differences between BAT‐Mfn2‐KO and WT mice. Acute cold exposure (4°C for 7 h) revealed an inability of BAT‐Mfn2‐KO mice to maintain body temperature after 3 h of exposure (Fig 1B and C). Indeed, at 6 h of exposure, 50% of BAT‐Mfn2‐KO female mice showed signs of torpor. This is consistent with the 34% reduction in thermogenic Ucp1 protein levels induced by Mfn2 loss in BAT (Fig 1D and E).

**Figure EV2:**
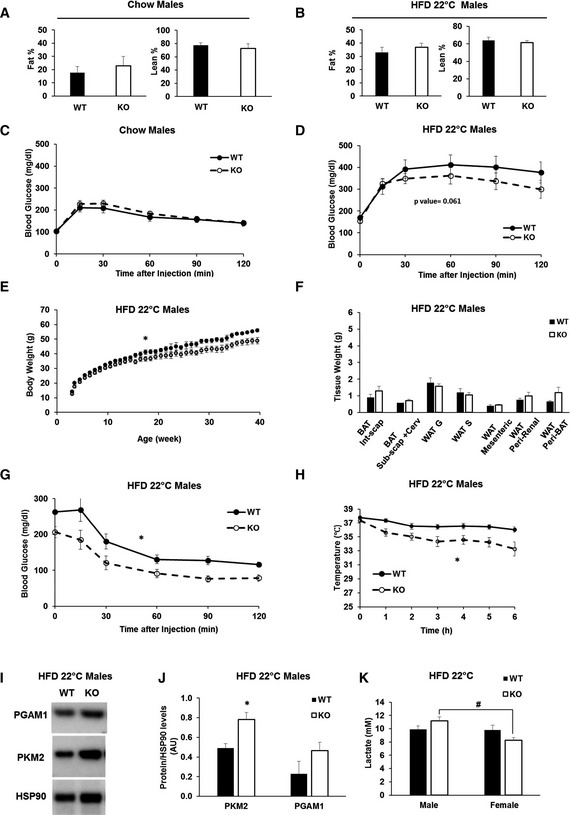
BAT‐Mfn2‐KO male mice are protected from HFD‐induced insulin resistance and increase BAT glycolytic capacity, despite showing the same body fat gain and being cold‐intolerant Body composition of *n* = 8–9 wild type (WT) and BAT‐Mfn2‐KO (KO) male mice (7 months) per group on chow diet. Bar graph represent average of % fat and % lean mass of total body weight ± SEM.Body composition of *n* = 4–6 wild type (WT) and BAT‐Mfn2‐KO (KO) male mice (7 months) per group on a HFD at ambient temperature (22°C). Bar graph represent average of % fat and % lean mass of total body weight ± SEM.Glucose tolerance test (GTT) on *n* = 11–16 wild type (WT) and BAT‐Mfn2‐KO (KO) male mice per group at 5 months old, fed chow diet. Values shown are average ± SEM.GTT on *n* = 10–13 wild type (WT) and BAT‐Mfn2‐KO (KO) male mice per group, fed a HFD at 22°C. Values shown are average ± SEM. Two‐way ANOVA test, WT vs. KO, *P* < 0.05.Body weight measurements of *n* = 8–9 wild type (WT) and BAT‐Mfn2‐KO (KO) male mice per group on HFD at an ambient temperature of 22°C (room temperature, RT) over 40 weeks. Values shown are average ± SEM. Two‐way ANOVA test, WT vs. KO, *P < 0.05.Quantification of WAT and BAT deposit weight isolated from *n* = 4–6 wild type (WT) and BAT‐Mfn2‐KO (KO) male mice per group fed a HFD at ambient temperature (22°C). Bar graphs represent average ± SEM. * represents significance using Student's *t*‐test, unpaired *P* < 0.05.Insulin tolerance tests (ITT) on *n* = 9–13 wild type (WT) and BAT‐Mfn2‐KO (KO) male mice per group, fed a HFD at ambient temperature (22°C). Values shown are average ± SEM. Two‐way ANOVA test, WT vs. KO, *P < 0.05.Body temperature measurements of wild type (WT) and BAT‐Mfn2‐KO (KO) male (*n* = 10–12) mice at 9 months old in HFD groups at ambient temperature. Values shown in both panels are means ± SEM. * represents significance using two‐way ANOVA test, WT vs. KO, *P* < 0.05.Representative Western blot measuring PGAM1, PKM2, and HSP90 in BAT total lysates from wild type (WT) and BAT‐Mfn2 KO (KO) males on HFD.Protein level quantification of PGAM1 and PKM2 normalized by HSP90 level, used as loading control. Bar graphs represent average ± SEM of proteins normalized to HSP90 from *n* = 3–4 mice per group of wild type (WT) and BAT‐Mfn2‐KO (KO) male mice. * represents significance using Student's *t*‐test, unpaired *P* < 0.05.Quantification of serum lactate from *n* = 3–6 male and *n* = 4–8 female mice per group of wild type (WT) and BAT‐Mfn2‐KO (KO) mice. Bar graphs represent average ± SEM of serum lactate (mM). ^#^ represents significance between male KO and female KO groups using Student's *t*‐test, unpaired *P* < 0.05.

Except for cold intolerance, BAT‐Mfn2‐KO mice had no visible phenotypic changes, exhibiting normal development and fertility and with similar values of body weight after 30 weeks under chow diet (Fig 1F and G). Body composition, measured as percentage of fat and lean body mass, was not different in BAT‐Mfn2‐KO mice (Appendix Fig S1A and Fig EV2A). Consistent with the NMR data, dissected WAT depots did not show any significant differences in mass between control (WT) and BAT‐Mfn2‐KO mice (Fig 1H and I). On the other hand, we detected increased BAT mass in BAT‐Mfn2‐KO mice (Fig 1H and I).

We further characterized BAT hypertrophy of BAT‐Mfn2‐KO by histology. H&E‐stained sections showed larger lipid droplets in Mfn2‐deleted BAT, without changes in the size of the rest of the cell (Fig 1J and K). These results are similar to the effects caused by Ucp1 deletion 10, 11, thermoneutrality 12, and diet‐induced obesity on BAT 4. This suggests that BAT hypertrophy in BAT‐Mfn2‐KO was mediated by lipid accumulation (lipohypertrophy) in chow diet‐fed mice.

### Deletion of Mfn2 in BAT remodels mitochondrial structure and function

**Figure 2:**
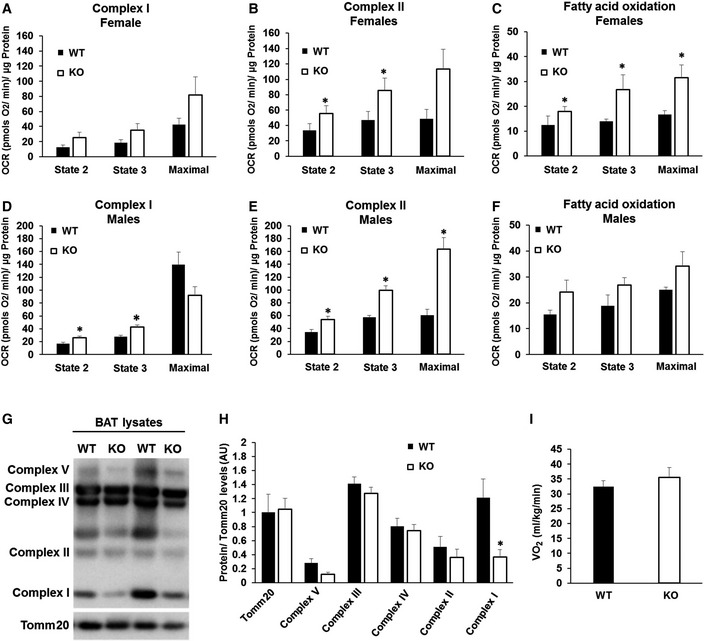
BAT mitochondria from BAT‐Mfn2‐KO mice show improved respiratory capacity and changes in respiratory complex expression levels A–CQuantification of oxygen consumption rates (OCR) in BAT isolated mitochondria from wild type (WT) and BAT‐Mfn2‐KO female mice under chow diet under the different respiratory states. State 2 quantifies respiration driven by proton leak (no‐ATP synthesis), state 3 quantifies respiration linked to maximal ATP synthesis, and maximal represents maximal electron transport chain activity induced by FCCP. Bar graphs represent average ± SEM for complex I‐driven respiration (pyruvate–malate, *n* = 4–5 mice per group) (A), complex II‐driven respiration (succinate−rotenone, *n* = 4–5 mice per group) (B), and fatty acid oxidation (palmitoyl carnitine−malate, *n* = 3 mice per group) (C). * represents significance using Student's *t*‐test, unpaired *P* < 0.05.D–FQuantification of OCR in BAT isolated mitochondria from wild type (WT) and BAT‐Mfn2‐KO male mice under chow diet under the different respiratory states. State 2 quantifies respiration driven by proton leak (no‐ATP synthesis), state 3 quantifies respiration linked to maximal ATP synthesis, and maximal represents maximal electron transport chain activity induced by FCCP. Bar graphs represent average ± SEM for complex I‐driven respiration (pyruvate–malate, *n* = 13–19 mice per group) (D), complex II‐driven respiration (succinate−rotenone, *n* = 13–19 mice per group) (E), and fatty acid oxidation (palmitoyl carnitine−malate, *n* = 2–8 mice per group) (F). * represents significance using Student's *t*‐test, unpaired *P* < 0.05.GRespiratory complex expression levels: Representative Western blot measuring complex I subunit Ndufb8, complex II Sdhb, complex III Uqcrc2, complex IV Cox1, complex V Atp5a, and outer mitochondrial membrane protein Tomm20 in BAT total lysate from wild type (WT) and BAT‐Mfn2‐KO female mice.HQuantification of the five complex subunits normalized by Tomm20 level measured as shown in (G). Bar graphs represent average ± SEM of Tomm20 expression values and complexes normalized to Tomm20 from *n* = 5–7 mice per group of wild type (WT) and BAT‐Mfn2‐KO female mice. * represents significance using Student's *t*‐test, unpaired *P* < 0.05.IOxygen consumption measurements in WT and BAT‐Mfn2‐KO anesthetized females under a chow diet 30 min after injection with the β3‐agonist CL‐316,243 (1 mg/kg). Bars represent average ± SEM of V_O2_ (ml/kg/min) consumed *n* = 4–7 mice per group.

To address the effects of Mfn2 deletion on mitochondrial fat oxidation and electron transport chain function in BAT, we measured oxygen consumption in isolated mitochondria under different fuels. Complex II‐driven respiration under state 2 (proton leak) and state 3 (coupled to maximal ATP synthesis capacity) was increased in BAT‐Mfn2‐KO mitochondria (Fig 2B and E). Complex I‐driven state 2 and 3 respiration was upregulated in Mfn2‐deleted BAT mitochondria from males (Fig 2D), while showing a trend to be increased in all states in females (Fig 2A). These increases were present despite the specific reduction in Ndufb8 protein levels, without changes in the levels of the remaining OXPHOS complexes (Fig 2G and H). Ndufb8 reduction might represent a decrease in molecules not assembled into functional supercomplexes/complex I, which has been shown to occur when coupled mitochondria use fatty acids as a fuel 13. When provided with fatty acids (palmitoyl carnitine), Mfn2‐deleted BAT mitochondria from females increased oxygen consumption in all respiratory states (Fig 2C), in contrast to the absence of significant changes in males (Fig 2F). No changes in coupling efficiency quantified by the respiratory control ratio (RCR) were observed (Appendix Fig S3A and B). Altogether, these results suggest that Mfn2 deletion *in vivo* remodels mitochondria to increase respiratory capacity coupled to ATP synthesis, at the expense of decreasing thermogenic Ucp1. Thus, in the absence of Mfn2, oxygen consumption in BAT mitochondria will be under a higher control of the cellular ATP/ADP ratio, rather than of thermogenic Ucp1 activity. To confirm these findings, we measured oxygen consumption rates in anesthetized WT and BAT‐Mfn2 KO female mice after β3‐adrenergic stimulation. As expected, BAT‐Mfn2‐KO mice had a similar increase in oxygen consumption in response to adrenergic stimulation (Fig 2I). Consequently, we conclude that in the absence of Mfn2, a decrease in Ucp1‐controlled mitochondrial fat oxidation (Fig 1D and E), is replaced by increased mitochondrial fat oxidation capacity controlled by ATP demand (Fig 2C).

Next, we tested the effect of Mfn2 deletion on mitochondrial morphology and mass within BAT. Electron microscopy revealed fragmented and less interconnected mitochondria in Mfn2‐deleted BAT, as shown by decreased aspect ratio and form factor and expected by the inhibition of mitochondrial fusion (Appendix Fig S2A–F). Total mitochondrial counts and average area were not changed in Mfn2‐deleted BAT (Appendix Fig S2E and F), confirming an absence of changes in mitochondrial mass as seen by Tomm20 staining (Appendix Fig S2G and H). On the other hand, we detected a reduction in mtDNA, showing a disconnection between mtDNA, mitochondrial mass, and mitochondrial protein levels (Appendix Fig S2I). This disconnection is characteristic of altered mitochondrial dynamics 14.

### Mfn2 deletion in BAT protects from diet‐induced obesity

**Figure 3:**
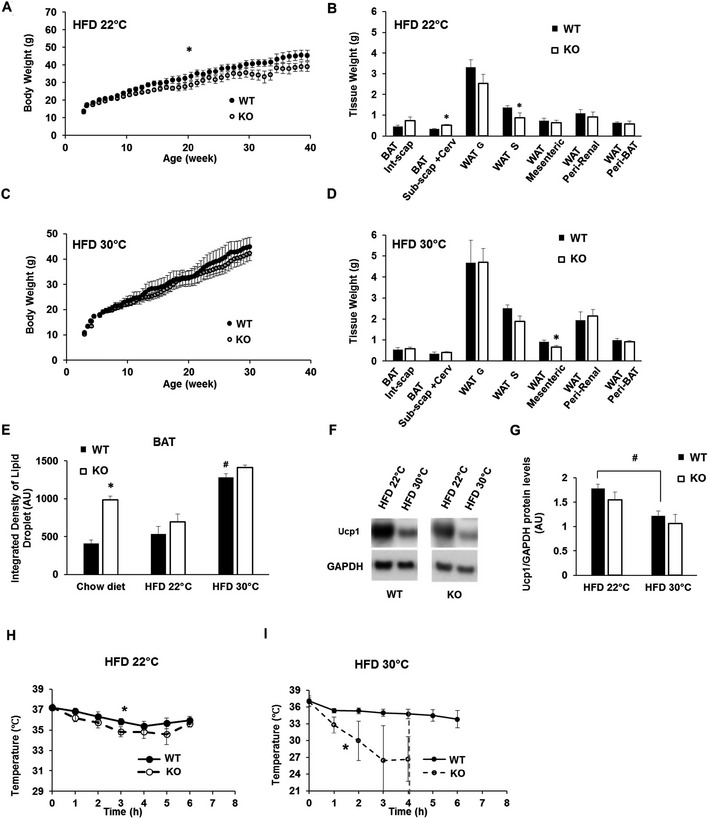
BAT‐specific deletion of Mfn2 in females protects from diet‐induced obesity, despite cold intolerance Body weight measurements of *n* = 8–10 wild type (WT) and BAT‐Mfn2‐KO (KO) female mice per group on HFD at an ambient temperature of 22°C (room temperature, RT) over 40 weeks. Values shown are average ± SEM. Two‐way ANOVA test, WT vs. KO,* *P* < 0.05.Quantification of WAT and BAT depots weight isolated from *n* = 4–8 wild type (WT) and BAT‐Mfn2‐KO (KO) female mice per group fed a HFD at ambient temperature (22°C). Bar graphs represent average ± SEM. * represents significance using Student's *t*‐test, unpaired *P* < 0.05.Body weight measurements of *n* = 5–7 wild type (WT) and BAT‐Mfn2‐KO (KO) female mice per group fed a HFD at thermoneutrality (30°C) over 30 weeks. Values shown are average ± SEM. No significant differences detected. Two‐way ANOVA test, WT vs. KO.Quantification of WAT and BAT depots weight of *n* = 5–7 wild type (WT) and BAT‐Mfn2‐KO (KO) female mice per group fed a HFD at thermoneutrality (30°C). Bar graphs represent average ± SEM. * represents significance using Student's *t*‐test, unpaired *P* < 0.05.Quantification of the lipid droplet integrated density from the BAT isolated from wild type (WT) and BAT‐Mfn2‐KO (KO) female mice fed a chow diet (*n* = 5) or a HFD at either 22°C (*n* = 5–6) or 30°C (*n* = 4–8). Values shown are average ± SEM. * represents significance WT vs. KO using Student's *t*‐test, unpaired *P* < 0.05. ^#^ represents significance WT chow diet vs. WT HFD 30°C using Student's *t*‐test, unpaired *P* < 0.05.Representative Western blot measuring Ucp1 and GAPDH (mitochondrial loading control) in BAT total lysates from wild type (WT) and BAT‐Mfn2‐KO (KO) female mice fed a HFD at 22°C or at 30°C.Protein level quantification of Ucp1 protein levels normalized to GAPDH. Bars represent average of Ucp1/GAPDH from *n* = 3–4 mice per group ± SEM. ^#^ represents significance using Student's *t*‐test, unpaired HFD 22°C vs. 30°C *P* < 0.05.Body temperature measurements before and during cold exposure (4°C) of wild type (WT) and BAT‐Mfn2‐KO (KO) female (*n* = 7–11 mice per group), at 9 months old, fed a HFD at ambient temperature. Values shown in both panels are means ± SEM. * represents significance using two‐way ANOVA test, WT vs. KO, *P* < 0.05.Body temperature measurements before and during cold exposure (4°C) of wild type (WT) and BAT‐Mfn2‐KO (KO) female mice (*n* = 5–7 mice per group), at 9 months old, fed a HFD at thermoneutral temperature. Values shown in both panels are means ± SEM. * represents significance using two‐way ANOVA test, WT vs. KO, *P* < 0.05. Red dotted line: At this time point all the KO mice were removed from the cold room due to severe cold intolerance.

To test the role of BAT‐Mfn2 protein in HFD‐induced obesity and insulin resistance, we fed control and BAT‐Mfn2‐KO mice a HFD for 40 weeks at either 22°C or thermoneutrality (30°C). Following 10 weeks of HFD at 22°C, BAT‐Mfn2‐KO females gained less body weight, an effect that was maintained through week 40 (Fig 3A). Dissection of adipose depots revealed that HFD‐fed BAT‐Mfn2‐KO females at 22°C had a significant decrease in their subcutaneous WAT (WAT S) and a trend toward reduced perigonadal WAT (WAT G) mass (Fig 3B). The differences in body weight in BAT‐Mfn2‐KO females at 22°C were not explained by a reduction in weekly HFD food intake (Appendix Fig S1B and C). These results were in marked contrast with BAT‐Mfn2‐KO male WAT depots, which were not reduced despite their lower total body weight (Fig EV2B, E and F). Therefore, absence of Mfn2 in BAT was not protecting from obesity in males, but rather reducing total lean mass. Interestingly, HFD decreased the difference in BAT lipohypertrophy between WT and BAT‐Mfn2‐KO females (Fig 1H vs. Fig 3B and D), with similar effect in males (Fig 1I vs. Fig EV2F). Accordingly, BAT from WT and BAT‐Mfn2‐KO females showed no changes in lipid droplet area when fed a HFD (Fig 3E). This observation is similar to the effect previously reported in Ucp1 KO mice fed a HFD, in which the differences in BAT lipohypertrophy between WT and Ucp1^−/−^ mice were eliminated by diet‐induced obesity 10.

A housing temperature of 30°C eliminates thermal stress in mice (thermoneutrality). This temperature is used to determine the effects of HFD feeding on obesity that are dependent on BAT‐Ucp1‐mediated energy expenditure 10. Thus, we tested the effects of thermoneutrality on the decreases in WAT mass detected in BAT‐Mfn2‐KO females fed a HFD at 22°C. Mfn2‐dependent differences in subcutaneous and perigonadal WAT under HFD feeding were almost eliminated by thermoneutrality (Fig 3A–D). In contrast, BAT‐Mfn2 deletion mildly, but significantly, reduced mesenteric WAT mass (Fig 3D). Therefore, thermoneutrality largely removed the protective effect induced by Mfn2 deletion on obesity.

We next determined the effects of thermoneutrality combined with HFD on BAT in the context of Mfn2 deletion. Thermoneutrality eliminated the reduction in Ucp1 expression induced by Mfn2 loss (Fig 3F and G), suggesting that BAT‐Mfn2‐KO mice were more sensitive to the adrenergic stimulation induced by HFD. Consistent with the reduction in energy demand associated to thermal stress, thermoneutrality induced an increase in lipid droplet size in BAT of WT HFD‐fed mice as compared to mice on the same diet but housed at 22°C. This phenomenon occurred without defects in BAT‐Mfn2‐KO mice, as shown by their similar lipid droplet size when compared to WT mice (Fig 3E).

Next, we tested whether diet‐induced obesity changed the ability of BAT‐Mfn2‐KO mice to sustain cold‐induced thermogenesis. In this context, HFD feeding at 22°C slightly improved cold tolerance in BAT‐Mfn2‐KO mice (Fig 3H vs. Fig 1B), but their tolerance to cold was still significantly lower than WT obese mice (Fig 3H). On the other hand, the combination of thermoneutrality and HFD caused severe cold intolerance in BAT‐Mfn2‐KO mice (Fig 3I), despite the restoration of Ucp1 to WT levels induced by thermoneutrality in Mfn2‐deleted BAT (Fig 3F and G). Consequently, these results suggest that in the absence of thermal stress, the adrenergic response induced by HFD is not sufficient to restore the thermogenic defect caused by Mfn2 deletion in BAT.

### Mfn2 deletion in BAT protects from insulin resistance induced by obesity

**Figure 4:**
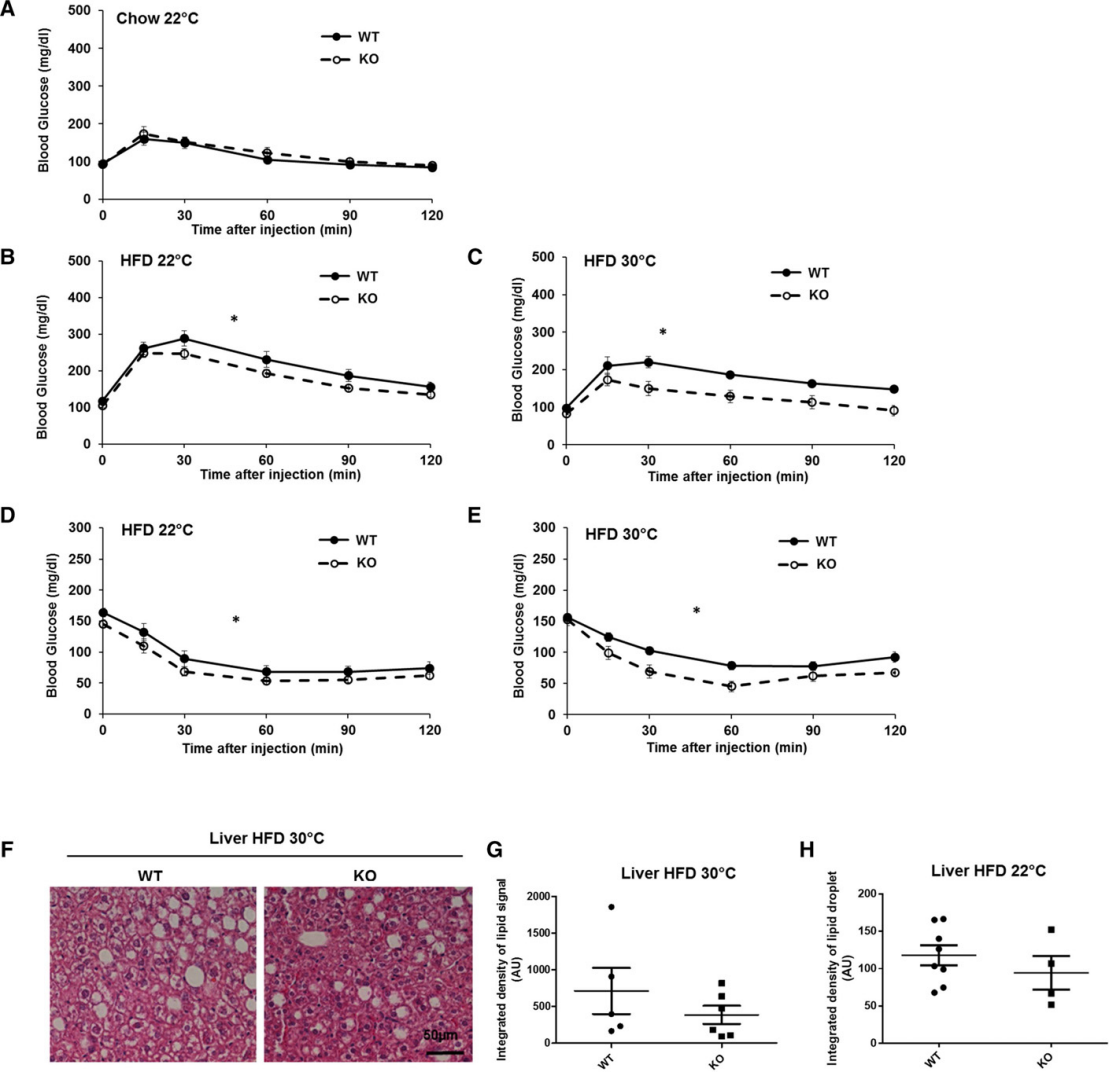
BAT‐specific deletion of Mfn2 prevents HFD‐induced insulin resistance Glucose tolerance test (GTT) on *n* = 5–7 wild type (WT) and BAT‐Mfn2‐KO (KO) female mice per group at 5 months old, fed chow diet.GTT on *n* = 10 wild type (WT) and BAT‐Mfn2‐KO (KO) female mice per group, fed a HFD at 22°C.GTT on *n* = 5–7 wild type (WT) and BAT‐Mfn2‐KO (KO) female mice per group, fed a HFD at thermoneutral temperature (30°C).Insulin tolerance tests (ITT) on *n* = 8–10 wild type (WT) and BAT‐Mfn2‐KO (KO) female mice per group, fed a HFD at 22°C.ITT on *n* = 5–7 wild type (WT) and BAT‐Mfn2‐KO (KO) female mice per group, fed a HFD at thermoneutral temperature (30°C). Glucose injection was required due to hypoglycemia 60 min after insulin injection to some BAT‐Mfn2‐KO mice.Representative images of H&E staining of the liver sections isolated from wild type (WT) and BAT‐Mfn2‐KO (KO) female mice, fed a HFD at thermoneutrality (30°C).Quantification of the integrated density of lipid droplet signal in liver sections from *n* = 5–6 wild type (WT) and BAT‐Mfn2‐KO (KO) female mice per group fed a HFD at thermoneutrality. Each dot represents a mouse and values shown are expressed as arbitrary units. Values shown are average ± SEM. Student's *t*‐test, unpaired *P* > 0.05.Quantification of the integrated density of lipid droplet signal in liver sections from *n* = 4–8 wild type (WT) and BAT‐Mfn2‐KO (KO) female mice per group fed a HFD at 22°C. Each dot represents a mouse and values shown are expressed as arbitrary units. Values shown are average ± SEM. Student's *t*‐test, unpaired *P* > 0.05.Data information: Values in panels (A–E) are average ± SEM. * represents significance using two‐way ANOVA test, *P* < 0.05.

We next tested the effects of BAT‐Mfn2 deletion on glucose tolerance in obese mice. Surprisingly, while thermogenesis was impaired, insulin sensitivity was improved. Intraperitoneal glucose tolerance tests (GTTs) in HFD fed mice showed that BAT‐Mfn2‐KO females were more glucose‐tolerant than WT, both at 22°C and at thermoneutrality (Fig 4B and C). These improvements were associated with significant increases in insulin sensitivity, as shown by intraperitoneal insulin tolerance tests (ITT) (Fig 4D and E). Male mice showed similar improvements in glucose handling (Fig EV2D and G). We observed no differences in fasting glucose levels (time 0, Fig 4D and E) and in liver steatosis (Fig 4F–H). As these two parameters are hallmarks of hepatic insulin resistance, these results show that BAT itself and likely other tissues contribute to improved insulin sensitivity in BAT‐Mfn2‐KO mice. Importantly, BAT‐Mfn2‐KO mice fed a chow diet did not show differences in glucose tolerance (Figs 4A and EV2C), demonstrating that metabolic improvement is a consequence of resistance to diet‐induced obesity and/or requires the component of HFD stimulating an adrenergic response in BAT 10.

Remarkably, the improvement in glucose tolerance and insulin sensitivity in BAT‐Mfn2‐KO was further amplified under thermoneutrality (Fig 4C and E). These results are in sharp contrast to the enhancement of cold intolerance induced by thermoneutrality in BAT‐Mfn2‐KO mice (Fig 3H and I). Consequently, these data demonstrate that BAT remodeling can protect from HFD‐induced insulin resistance independently of the ability to sustain body temperature after cold exposure. Furthermore, they show that protection from insulin resistance driven by primary changes in BAT can be inversely correlated with cold tolerance.

### Mfn2 deletion induces gender‐specific mitochondrial and metabolic remodeling of BAT in response to obesity

To determine whether obesity‐induced BAT remodeling was different in Mfn2‐deleted BAT, we characterized mitochondria from BAT‐Mfn2‐KO males and females fed a HFD at 22°C. We measured OXPHOS complex levels, as well as their mitochondrial respiratory function and morphology.

### Response of BAT‐Mfn2‐KO female to obesity: increased mitochondrial coupling of fat oxidation to ATP synthesis

**Figure 5:**
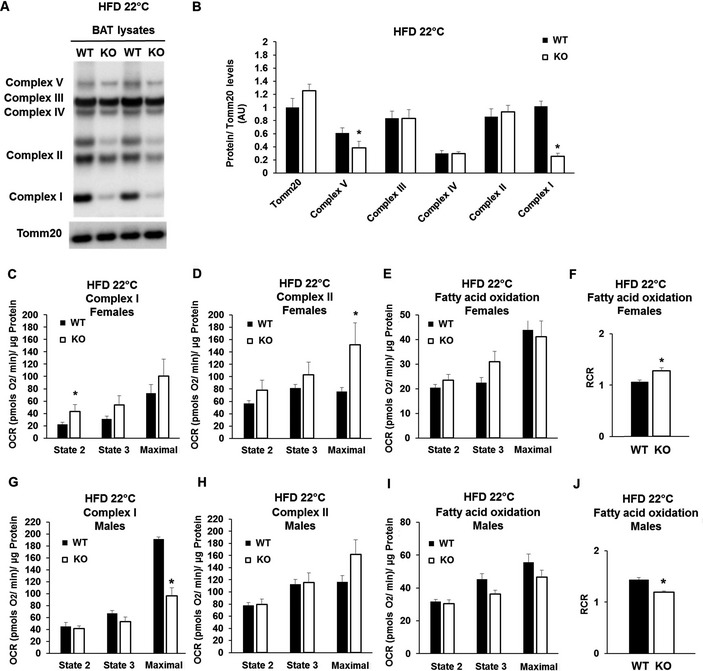
Mfn2 deletion increases the efficiency of ATP synthesizing respiration of BAT mitochondria oxidizing fat in obese females, but not in males ARepresentative Western blot analysis measuring complex I subunit Ndufb8, complex II Sdhb, complex III Uqcrc2, complex IV Cox1, complex V Atp5a, and outer mitochondrial membrane Tomm20 in BAT total lysate from wild type (WT) and BAT‐Mfn2 KO (KO) female mice on high fat diet (HFD).BProtein level quantification of Tomm20 per microgram of total protein loaded, and the five complex subunits normalized by Tomm20 level, used as loading control. Bar graphs represent average ± SEM of Tomm20 expression values and complexes normalized to Tomm20 from *n* = 4–8 mice per group of wild type (WT) and BAT‐Mfn2‐KO (KO) female mice. * represents significance using Student's *t*‐test, unpaired *P* < 0.05.C–EQuantification of oxygen consumption rates (OCR) in BAT isolated mitochondria from wild type (WT) and BAT‐Mfn2‐KO (KO) female mice fed a HFD at room temperature 22°C. State 2 quantifies respiration driven by proton leak (no‐ATP synthesis), state 3 quantifies respiration linked to maximal ATP synthesis, and maximal represents maximal electron transport chain activity induced by FCCP. Bar graphs represent average ± SEM for complex I‐driven respiration (pyruvate–malate, *n* = 4–8 mice per group) (C), (succinate−rotenone, *n* = 4–8 mice per group) (D), and fatty acid oxidation (palmitoyl carnitine−malate, *n* = 4–6 mice per group) (E). * represents significance using Student's *t*‐test, unpaired *P* < 0.05.FQuantification of the respiratory control ratio (RCR, state 3/state 2) measured in isolated BAT mitochondria from *n* = 4–6 wild type (WT) and BAT‐Mfn2‐KO female mice per group fed a high fat diet at 22°C and using fatty acids (palmitoyl carnitine−malate) as fuels for oxidation. Bar graphs represent average ± SEM. * represents Student's *t*‐test, unpaired WT vs. KO *P* < 0.05.G–IQuantification of OCR in BAT isolated mitochondria from wild type (WT) and BAT‐Mfn2‐KO male mice fed a HFD at room temperature 22°C. State 2 quantifies respiration driven by proton leak (no‐ATP synthesis), state 3 quantifies respiration linked to maximal ATP synthesis, and maximal represents maximal electron transport chain activity induced by FCCP. Bar graphs represent average ± SEM for complex I‐driven respiration (pyruvate–malate, *n* = 3–6 mice per group) (G), (succinate−rotenone, *n* = 3–6 mice per group) (H), and fatty acid oxidation (palmitoyl carnitine−malate, *n* = 2–5 mice per group) (I). * represents significance using Student's *t*‐test, unpaired *P* < 0.05.JQuantification of the respiratory control ratio (RCR, state 3/state 2) measured in isolated BAT mitochondria from *n* = 2–5 wild type (WT) and BAT‐Mfn2‐KO (KO) male mice per group fed a HFD at 22°C and using fatty acids (palmitoyl carnitine−malate) as fuels for oxidation. Bar graphs represent average ± SEM. * represents Student's *t*‐test, unpaired WT vs. KO *P* < 0.05.

Mfn2‐deleted BAT mitochondria isolated from females fed a HFD at 22°C showed enhanced respiration in all states, driven by either complex I or complex II. Respiratory increases only reached statistical significance in the maximal respiration state driven by complex II and in state 2 driven by complex I, both around twofold increase (Fig 5C and D). Interestingly, in the case of mitochondrial respiration using fatty acids as a fuel, BAT‐Mfn2‐KO female mitochondria showed increased coupled respiratory efficiency, as shown by the increase in the respiratory control ratio (RCR, state 3/state 2) (Fig 5F), as a result of a 30% increase in state 3 respiration (Fig 5E). These results suggest that under HFD, Mfn2‐deleted BAT mitochondria are remodeled to increase ATP production, but in this case through an increase in RCR.

To better understand these changes in mitochondrial function, we looked at mitochondrial protein expression. As compared to chow diet, under HFD deletion of Mfn2 lead to a larger (75%) decrease in complex I subunit Ndufb8 protein (Fig 5A and B), consistent with a shift toward coupled fatty acid oxidation 13. The rest of OXPHOS complexes did not show differences (Fig 5A and B) with the exception of complex V, which was reduced 36% (Fig 5A and B). The reduction in complex V likely represents mitochondrial remodeling, as state 3 respiration and RCR values are increased (Fig 5E and F). We analyzed mitochondrial morphology in BAT‐Mfn2‐KO females in response to HFD at 22°C by electron microscopy and quantified mtDNA levels. The reduction in mitochondrial form factor, a feature of fragmented and more spherical mitochondria, was maintained in BAT‐Mfn2‐KO females fed a HFD (Appendix Fig S4A–C). However, HFD caused mild swelling of BAT‐Mfn2‐KO mitochondria, when compared to WT (measured as increased mitochondria area, Appendix Fig S4D), but cristae density was not changed (Appendix Fig S4E). The reduction in mtDNA in BAT‐Mfn2‐KO females observed in chow diet was maintained under HFD at 22°C (Appendix Fig S4F).

### Response of BAT‐Mfn2‐KO male to obesity: decreased maximal respiratory capacity and reduced coupling, with increased glycolytic capacity

In marked contrast to BAT‐Mfn2‐KO females, BAT‐Mfn2‐KO male mice were not protected from HFD‐induced fat gain in WAT (Fig EV2B and F). This suggested that BAT function in the BAT‐Mfn2‐KO male responded differently to obesity. To address this possibility, we measured mitochondrial fat oxidation and electron transport chain function in isolated mitochondria from BAT. Complex I‐ and II‐driven respiration under state 2 and state 3 was unaffected. This result indicates that unlike female mitochondria, ATP synthesis efficiency measured by the RCR was not improved (Fig 5G and H, and Appendix Fig S3C). Moreover, when respiration used fatty acids as fuels, Mfn2‐deleted BAT mitochondria from obese males showed decreased RCR values, indicating that ATP synthesis efficiency was decreased (Fig 5I and J). This is in sharp contrast to BAT‐Mfn2‐KO obese females (Fig 5F).

The most striking difference between obese BAT‐Mfn2‐KO males and females was a dramatic decrease in maximal respiratory capacity driven by complex I respiration (Fig 5G). This capacity may reflect the integrated effect of Mfn2 deletion on the activity of complex I as well as pyruvate–malate transport across the mitochondria and TCA cycle dehydrogenases activity. Therefore, the maximal capacity to oxidize pyruvate from glucose is reduced in Mfn2‐deleted BAT mitochondria from males.

Given that BAT‐Mfn2‐KO obese male mice cleared glucose more efficiently during an ITT (Fig EV2G), we hypothesized that BAT from BAT‐Mfn2‐KO males had their metabolism re‐wired to support glucose disposal by increasing glycolysis to lactate. To this end, we measured lactate levels in the serum of BAT‐Mfn2‐KO males fed a HFD. Consistent with increased BAT glycolytic capacity, we detected an increase in serum lactate levels in BAT‐Mfn2‐KO obese males, when compared both to WT obese males and BAT‐Mfn2‐KO obese females (Fig EV2K). Protein levels of the glycolytic enzyme, PKM2, were markedly increased in Mfn2‐deleted BAT from obese males (Fig EV2I and J). Therefore, these data support that Mfn2 deletion in BAT from males induces a metabolic remodeling favoring glycolytic capacity, in response to obesity.

### Metabolic improvement in BAT‐Mfn2‐KO females is associated with increased lipid oxidation without enhanced heat production *in vivo*

**Figure 6:**
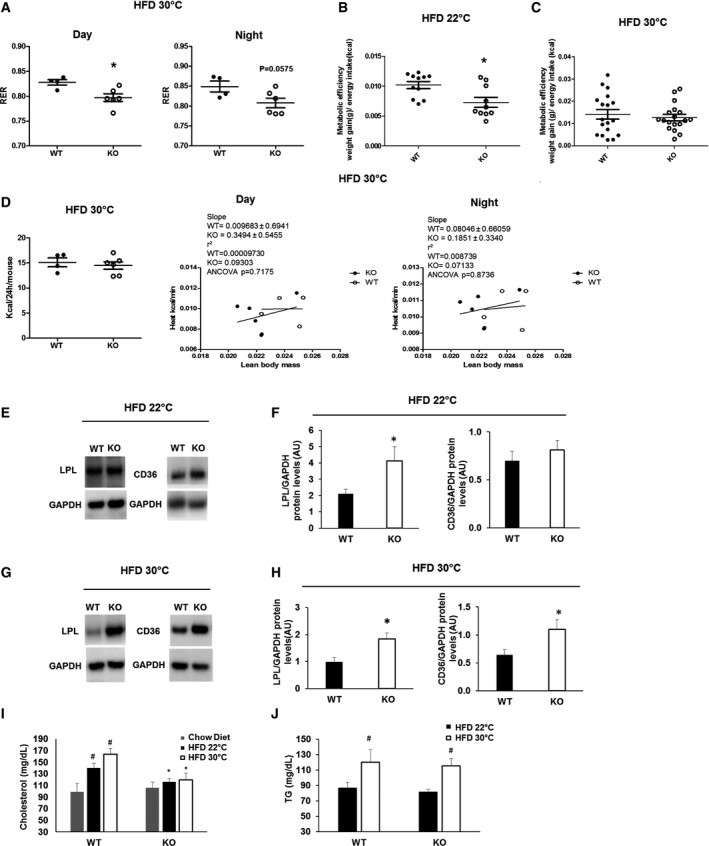
Increased capacity for fatty acids oxidation, coupled respiration and lipid import, accompanied by reduced serum lipids, in obese BAT‐Mfn2‐KO female mice AQuantification of the RER measurements at both light and dark cycles for *n* = 4–6 wild type (WT) and BAT‐Mfn2‐KO (KO) female mice per group fed a HFD at thermoneutrality (30°C). Values are calculated as the ratio of V_CO2_ to V_O2_ produced and consumed by the mice, respectively. Each dot represents a mouse. Values shown are average ± SEM. * represents significance using Student's *t*‐test, unpaired *P* < 0.05.B, CMetabolic efficiency of wild‐type (WT) and BAT‐Mfn2‐KO (KO) female mice fed a HFD at room temperature, 22°C (*n* = 10–11 mice per group) (B) and at thermoneutrality (*n* = 5–7 mice per group) (C). Values are calculated as gram of body weight gained per Kcal of energy intake. Values shown in both panels are mean ± SEM. * represents significance using Student's *t*‐test, unpaired *P* < 0.05.DQuantifications of mice whole‐body energy expenditure indicated as Kcal/24 h/mouse for *n* = 4–6 control and BAT‐Mfn2‐KO female mice per group under HFD at thermoneutrality (30°C), and multiple regression analysis of energy expenditure correlated to lean body mass for the same control and BAT‐Mfn2‐KO female mice under HFD at 9 months old in light and dark cycles. Each dot represents a mouse in all panels. Values shown are average ± SEM. Student's *t*‐test unpaired and ANCOVA test were used respectively.ERepresentative Western blot measuring lipoprotein lipase (LPL), CD36, and GAPDH on BAT total lysates from wild type (WT) and BAT‐Mfn2‐KO (KO) female mice fed a high fat diet (HFD) at 22°C.FProtein level quantification of LPL and CD36 protein levels normalized to their corresponding loading control (GAPDH). Bars represent average of LPL/GAPDH from *n* = 3–8 female mice per group ± SEM. * represents Student's *t*‐test, unpaired WT vs. KO *P* < 0.05.GRepresentative Western blot measuring LPL, CD36, and GAPDH on BAT total lysates from wild type (WT) and BAT‐Mfn2‐KO (KO) female mice fed a high fat diet (HFD) at 30°C.HProtein level quantification of LPL and CD36 protein levels normalized to their corresponding loading control (GAPDH). Bars represent average of LPL/GAPDH from *n* = 5–7 female mice per group ± SEM. * represents Student's *t*‐test, unpaired WT vs. KO *P* < 0.05.IQuantification of serum cholesterol levels of wild type (WT) and BAT‐Mfn2‐KO (KO) female mice fed a chow (*n* = 7 per group) or a high fat diet at 22°C (*n* = 7–11 per group) or 30°C (*n* = 4–7 per group). Bars represent average of serum cholesterol levels (mg/dL) ± SEM. * and ^#^ represent Student's *t*‐test, unpaired WT vs. KO (*) and HFD 22°C or HFD 30°C vs. chow diet (^#^) *P* < 0.05.JQuantification of serum triglyceride (TG) levels of wild type (WT) and BAT‐Mfn2‐KO (KO) female mice fed a high fat diet at 22°C (*n* = 7–11 per group) or 30°C (*n* = 5–7 per group). Bars represent average of serum TG levels (mg/dL) ± SEM. ^#^ represents Student's *t*‐test, unpaired HFD 22°C vs. HFD 30°C *P* < 0.05.

Our next set of experiments aimed to test whether improved glucose handling in diet‐induced obese BAT‐Mfn2‐KO female mice at thermoneutrality could be explained by enhanced heat production and/or changes in nutrient preference in the absence of severe or mild thermal stress. To this end, metabolic cages were used to quantify energy expenditure by indirect calorimetry and determine nutrient preference for oxidative metabolism (glucose vs. fatty acids), through quantification of the respiratory exchange ratio (RER). BAT‐Mfn2‐KO female mice fed a HFD at thermoneutrality showed lower RER values, indicating higher preference and capacity for fatty acid oxidation over glucose (Fig 6A). Measurements of metabolic efficiency 10 show that BAT‐Mfn2‐KO females gained less weight per Kcal energy intake of HFD ingested under thermal stress (22°C), but not at thermoneutrality (Fig 6B and C).

BAT‐Mfn2‐KO females fed HFD at thermoneutrality showed no differences in heat generation, measured by indirect calorimetry and calculated as the correlation between heat generation and lean mass (Fig 6D). Accordingly, we did not detect any significant changes in locomotor activity of BAT‐Mfn2‐KO mice (Appendix Fig S1D). Consequently, these data together with the mitochondrial respiratory profile suggest that resistance to obesity and better insulin sensitivity induced by Mfn2 deletion in BAT is linked to increased coupled fatty acid oxidation in BAT and, possibly, in other tissues.

To test for the possibility that Mfn2 deletion in BAT results in expansion of beige cells, we measured protein levels of creatine kinase and Ucp1 mRNA within scWAT, as markers of beige adipocyte mass. We only detected an insignificant trend to an increase in Ucp1 mRNA levels in scWAT from BAT‐Mfn2‐KO females fed a HFD, at both 22°C and thermoneutrality (Appendix Fig S5A). No changes in creatine kinase levels and total mitochondrial mass measured as Tomm20 levels were detected (Appendix Fig S5B–F). Another possibility explaining increased insulin sensitivity in BAT‐Mfn2‐KO females at thermoneutrality could be through secretion of BAT “adipokines”, such as FGF21, which could decrease RER by promoting hepatic fat oxidation. No differences were detected in circulating FGF21 between WT and BAT‐Mfn2‐KO females fed a HFD at 22°C or at thermoneutrality (Appendix Fig S5G). Consistent with these findings, levels of hepatic steatosis measured by histology were similar (Fig 4F–H).

Next we tested whether BAT‐Mfn2‐KO at thermoneutrality promotes insulin sensitivity by further enhancing circulating lipids clearance in BAT, as previously shown 15. To address this hypothesis, we measured lipoprotein lipase (LPL) and CD36, major mediators of circulating fat uptake into BAT 15. Both CD36 and LPL protein levels were nearly doubled in BAT‐Mfn2‐KO females fed a HFD at thermoneutrality (Fig 6G and H). On the other hand, at 22°C, only LPL was significantly increased in BAT‐Mfn2‐KO obese females (Fig 6E and F). Indeed, these increases were associated with a reduction in circulating cholesterol levels in BAT‐MFN2‐KO obese females (Fig 6I). However, no significant changes in the thermoneutrality‐induced steady‐state levels of circulating TG were detected between WT and BAT‐Mfn2‐KO obese females (Fig 6J).

## Discussion

We had previously demonstrated that mitochondrial fragmentation, induced by cold exposure in BAT, might represent an approach to increase fat oxidation 9. Therefore, selectively inducing mitochondrial fragmentation in BAT could be a strategy mimicking Ucp1 activation and a potential therapy for obesity, which would bypass the need for adrenergic stimulation. However, the role of Mfn2 in obesity‐induced BAT remodeling and the effect of chronically reducing Mfn2 in BAT *in vivo* have not been explored.

Here we present a perturbation of brown adipocyte mitochondrial dynamics that enhances BAT capacity to negate the metabolic derangements associated with diet‐induced obesity, while impairing thermogenic capacity in response to cold *in vivo*. Our study identified for the first time a role for Mfn2 in the response of BAT to obesity and in a gender dependent manner. While in obese females deletion of Mfn2 results in increased coupled respiratory efficiency of BAT mitochondria oxidizing fat, Mfn2 deletion in obese males leads to reduced maximal complex I‐driven respiratory capacity. This male‐specific effect is accompanied by increased expression of the glycolytic enzyme PKM2 in BAT and by increased serum lactate levels *in vivo*. Whichever the response to Mfn2 deletion, it provides a superior capacity to prevent HFD‐induced insulin resistance. This is surprising, given that the physiological response to HFD feeding is an upregulation of Mfn2 and that deletion of Mfn2 causes cold intolerance. Our findings that Mfn2 is essential for proper thermogenic response to cold exposure support the notion that Mfn2 induction is part of the global increase in adrenergic stimulation induced by thermal stress and by diet‐induced obesity 8, 10. However, it raises doubts whether increasing thermogenic capacity has evolved as a compensatory mechanism to protect from obesity‐induced insulin resistance. Indeed, it is confirming that BAT has mechanisms to counteract obesity and insulin resistance separated from its role in cold‐induced thermogenesis 10. Furthermore, it suggests that BAT lipohypertrophy induced by obesity can be a regulated response protecting from insulin resistance 5, which has higher efficiency with Mfn2 deleted.

As independent studies previously reported in liver, muscle, and pancreatic beta‐cells, our data demonstrate that increased storage of lipids does not unequivocally represent a state of decreased mitochondrial oxidative capacity in BAT. Storage of lipids is energetically costly and ATP‐demanding lipid cycling is actively and constantly occurring, as lipid droplets are dynamic. For example, accumulation of lipid droplets in beta‐cells in obese subjects is associated with increased mitochondrial oxidative function due to lipid cycling. The increased ATP expenditure is thought to play a role in preventing the detrimental effects of excess lipids 16. In obesity‐induced steatosis, lipid accumulation in the liver is associated with an increase in both lipid utilization and mitochondrial respiration in hepatocytes 17. A third example, not linked to obesity, is the accumulation of lipid droplets in athlete's muscles, which show a marked increase in mitochondrial respiratory capacity 18. Our results suggest that similar to the above examples, the increased respiratory capacity in Mfn2‐deficient BAT supports the extra ATP production required to support lipohypertrophy.

Interestingly, Mfn2 and Ucp1 show striking similarities in their effects on BAT response to cold and obesity. Firstly, Mfn2 expression in BAT is upregulated by the same conditions inducing Ucp1 expression: HFD feeding and β3‐agonist adrenergic treatment 8. Secondly, both proteins are required to maintain body temperature after acute cold exposure of mice housed at 22°C and at thermoneutrality. Thirdly, ablation of either Ucp1 or Mfn2 changes the overall metabolic response to diet‐induced obesity in the absence of thermal stress. However, the specifics of this metabolic response are where the large differences between the two mouse models emerge. Loss of Mfn2 protects from diet‐induced obesity only at 22°C and from insulin resistance at both 22°C and thermoneutrality. In addition, protection from HFD‐induced insulin resistance in BAT‐Mfn2‐KO females is larger at thermoneutrality. This is in contrast to the Ucp1‐KO mice, where Ucp1 deletion causes obesity exclusively at thermoneutrality, without changes in body weight at 22°C. We suggest that these differences are mediated by metabolic and mitochondrial remodeling induced by Mfn2 deletion but not by Ucp1 deletion in BAT. Ucp1 deletion has no effects on the electron transport chain activity or fatty acid oxidation capacity coupled to ATP synthesis, while Mfn2 loss of function has been demonstrated to induce changes in nutrient oxidation, electron transport chain activity, and insulin signaling in other cell types 19, 20. In the case of obese females, we report that BAT‐Mfn2 deletion favors coupling fatty acid oxidation to ATP synthesis and in males supports glycolysis to lactate. Our data support that when mitochondrial oxidative function in BAT is remodeled to be driven by the ATP demand and controlled by the ATP/ADP ratio, it impinges on the mouse ability to sustain cold‐stimulated thermogenesis.

Whether the observed shift from uncoupled to coupled respiration in BAT‐Mfn2‐KO mitochondria is sufficient to explain their severe cold intolerance still remains an open question. Remarkably, restoration of Ucp1 levels in BAT‐Mfn2‐KO mice by HFD feeding did not prevent the observed shift from uncoupled to coupled respiration in Mfn2‐deleted mitochondria. This shows that the reduction in Ucp1 expression is not sufficient to explain this shift, nor the thermogenic defect induced by Mfn2 deletion in BAT.

As Mfn2 is primarily deleted in BAT of BAT‐Mfn2‐KO mice, their cold intolerance is unequivocally caused by a BAT‐specific manipulation and, consequently, it can be expected to be exclusively a result of BAT mitochondrial remodeling. Exclusivity is strongly supported by the absence of changes in heat generation and physical activity in obese BAT‐Mfn2‐KO mice under thermoneutrality, the conditions in which BAT pathways related to cold exposure are shut down. Consequently, primary defects in other tissues would largely contribute to heat generation and physical activity at thermoneutrality, and therefore, the results we obtained exclude this possibility. However, we cannot exclude the existence of an alternative mechanism by which deletion of Mfn2 in BAT promotes the secretion of inhibitory factors leading to decreased shivering and/or thermogenic function in muscle, only apparent during acute cold exposure. We think that these specific questions describing elusive molecular mediators responsible for a potential crosstalk between BAT and muscle during acute cold exposure are beyond this study, but we can conclude that BAT‐Mfn2‐KO mice can be used as a model to test these pathways.

Subcutaneous WAT, containing large numbers of beige adipocytes, was the only WAT deposit largely decreased in HFD‐fed BAT‐Mfn2‐KO females under mild thermal stress (22°C). These results suggested that a compensatory recruitment of beige adipocytes in scWAT could be explaining decreased scWAT mass. However, we did not detect changes in beige adipocyte recruitment in BAT‐Mfn2‐KO mice. Furthermore, changes in scWAT mass alone cannot explain improved protection from insulin resistance at thermoneutrality, when beige fat is mostly inactive 21, 22.

Accordingly, another established mechanism through which BAT function protects from obesity‐induced insulin resistance is the capacity of BAT to clear lipids from the circulation. LPL and CD36 were shown to play a major role in lipid clearance mediated by BAT 15. We detected an increase in LPL expression induced by Mfn2 loss in females fed a HFD at 22°C. Furthermore, at thermoneutrality, BAT‐Mfn2‐KO females showed even higher levels of LPL and CD36. These results demonstrate that increased lipid accumulation in BAT in obesity is a regulated process, modulated by thermal stress, and not exclusively associated with mitochondrial dysfunction or Ucp1 reduction 4. Indeed, lipid handling and storage is energetically expensive, requiring 2 ATP per each FFA activated with CoA. As a consequence, we suggest that mitochondrial coupling in females, and anaerobic glycolysis in males, is recruited to cover increased ATP demand to maintain BAT lipohypertrophy in the context of Mfn2 deletion. The need for this extra ATP in BAT in the absence of Mfn2 might shut down thermogenic/uncoupling pathways, resulting in cold intolerance.

A recent report by Boutant *et al* 23 describes a mouse model harboring Mfn2 deleted in both white and brown adipocytes (adipo‐Mfn2‐KO). Similar to the BAT‐Mfn2‐KO mice we describe here, the adipo‐Mfn2‐KO show defective thermogenesis, BAT lipohypertrophy and improved glucose tolerance after diet‐induced obesity 23. Combining the results from the two models, we suggest that these phenotypes likely stem from the activity of Mfn2 in the BAT. However, there are some important differences between the mouse models that are likely explained by the deletion of Mfn2 in white adipocytes 23. The first difference is the reduction in BAT mitochondrial respiratory function reported in the adipo‐Mfn2‐KO males fed a control diet. In the BAT‐Mfn2‐KO mice, we detect a decrease in maximal respiratory capacity in BAT mitochondria only in males fed a HFD, but not in females. These results suggest that reduced mitochondrial respiratory capacity in mice with Mfn2‐deleted BAT and fed a low fat diet requires at least three components: deletion of Mfn2 in WAT, being a male and mild thermal stress (22°C). Indeed, when adipo‐Mfn2‐KO males are housed under thermoneutrality, they still increase respiration in response to beta‐3‐adrenergic stimulation as wild‐type mice and their reduction in oxygen consumption is almost eliminated 23.

In summary, our study demonstrates that BAT‐Mfn2 deletion protects from insulin resistance induced by obesity, while impairing thermogenesis. Therefore, adaptation to cold and adaptation to obesity may represent two independent tasks that under some circumstances may prove to be conflicting.

## Materials and Methods

### Mouse models and tissue collection

Ucp1‐cre^(+/−)^ mice were provided by Dr. Aprahamian's laboratory and generated in Dr. Evan Rosen laboratory in C57Bl6/J background, currently in Jackson laboratories (Jax Stock No 024670). Mfn2loxP mice were provided by Dr. David Chan and generated in mixed C57Bl6J/129 background 14. Ucp1‐cre transgenic and Mfn2^flox/flox^ mice were crossed to generate Ucp1‐cre^+/−^ Mfn2^flox/flox^ (BAT‐Mfn2‐KO) and Ucp1‐cre^−/−^ Mfn2^flox/flox^ (control) mice. Different white adipose tissue depots (gonadal, subcutaneous, mesenteric, peri‐renal, and peri‐brown adipose tissue) as well as BAT depots (interscapular and subscapular plus cervical) were collected separately, weighed, and subsequently divided in order to provide uniform tissue samples. All experiments were approved by the Institutional Animal Care and Use Committee at Boston University. Mice were kept under a 12:12‐h dark–light period and provided with water and food *ad libitum*. Mice were divided to three groups at age 4–5 weeks depending on the room temperature and the provided diet. Group one were fed a standard chow diet (Harlan) and housed at room temperature (RT) (22°C), group two were fed a high fat diet (HFD, 45 kcal % fat) from Research Diets (catalogue # D12451) and housed at room temperature (22°C), and group three were fed HFD and housed at thermoneutral temperature (30°C).

### Thermogenesis and acute cold exposure

Subcutaneous, biocompatible, and sterile microchip transponders (Bio Medic Data Systems, Seaford, DE, USA) were implanted in male and female Ucp1‐cre^(−/−)^ Mfn2^flox/flox^ and Ucp1‐cre^(+/−)^ Mfn2^flox/flox^ mice in all three groups at least 2 days prior to experimentation. On the day of the experiment, mice were housed singly in pre‐chilled cages at 4°C with free access to water. Body temperature was assessed hourly for 6–8 h using a wireless reader system (Bio Medic Data Systems).

### Glucose tolerance test and insulin tolerance tests

Animals were fasted overnight with water available for GTT and for 6 h for ITT, and then blood glucose levels (fasting blood glucose) were determined before intraperitoneal (IP) injection of glucose (1 g/kg weight) or insulin (Humulin, 1 mU/g weight). Blood glucose levels are measured at different time points (15, 30, 60, 90, and 120 min) post‐injection using Accu‐Chek glucose strips and glucometer (Roche).

### Food intake, body weight, and metabolic chambers

Mice were weighted weekly, as well as fresh food added and remaining after 7 days. Intake was calculated by subtracting food added from food remaining in the food hopper and the cage. In the case of metabolic chambers, 2–3 days of adaptation to the cage was performed and then measurements were recorded for 2–3 days. Metabolic parameters were determined using indirect calorimetry in metabolic cages (Oxymax Comprehensive Lab Animal Monitoring System, Columbus Instruments, Columbus, OH, USA). The gas flow rate was 0.5 l/min. The instrument monitored the amount of oxygen inhaled (V_O2_) and the amount of carbon dioxide exhaled (V_CO2_) for 1 min every 18 min and continuously monitored the amount of activity on *x* (long)‐, *y* (short)‐, and *z* (vertical)‐axes. Total activity counted the number of times a laser beam was broken; ambulatory activity counted the number of times adjacent beams were broken and therefore did not register movement due to breathing, grooming, or scratching. RER was calculated with CLAX software (Columbus Instruments) as V_CO2_/V_O2_. Heat production (or energy expenditure) in cal/min was derived from the Lusk equation: (3.815 + 1.232 × RER) × V_O2_ with V_O2_ in ml/min. The system was calibrated with gas of a known percentage of oxygen and carbon dioxide before every experiment. The response to the β3 adrenergic agonist CL‐316,243 (1 mg/kg) was measured by recording whole‐body oxygen consumption in anesthetized mice (pentobarbital, 60 mg/kg), placed in metabolic chambers at thermoneutrality for 30 min. Then, CL‐316,243 (1 mg/kg) was injected subcutaneously and oxygen consumption was recorded for an additional 30 min.

### NMR and tissue collection

Fat mass and lean mass were acquired using the 5 G EchoMRI‐700 (EchoMRI, LLC, Houston, TX, USA). Mice were assessed in the fed state. The machine was calibrated with canola oil with known fat composition before every experiment.

### Mitochondrial isolation

Isolation buffer (SHE buffer) contains 250 mM sucrose, 5 mM HEPES, 2 mM EGTA, BSA 2% (pH = 7.2). Isolated BAT was rinsed and minced in ice‐cold PBS. Tissue pieces were transferred to the glass‐teflon dounce homogenizer containing SHE + BSA buffer and 9–10 strokes were performed to homogenize the tissue using the teflon pestle. The homogenate was centrifuged at 900 × *g* for 10 min at 4°C. This step was repeated with the supernatant. The resulting supernatant was centrifuged at 9,000 × *g* for 10 min at 4°C. The pellet was washed once and then re‐suspended in SHE without BSA. Protein content was measured by BCA.

### Respirometry measurements

Four micrograms of mitochondrial protein fractions were loaded per well for complex I‐driven respiration (pyruvate + malate) and 2 μg for complex II‐driven respiration (succinate + rotenone) in 25 μl of mitochondrial assay solution (MAS) per Seahorse XF96 well. The plate was centrifuged at 4°C, 5 min at 2,000 *g*. Then, 110 μl of MAS with the respective fuels was carefully added per well, on top of the 25 μl centrifuged. Before the respirometry assay within the XF96, the plate was warmed at 37°C for 4 min. MAS buffer contains 100 mM KCl, 10 mM KH_2_PO_4_, 2 mM MgCl_2_, 5 mM HEPES, 1 mM EGTA, 0.1% BSA, and 1 mM GDP (pH 7.2). Pyruvate is used at 5 mM, malate 5 mM, succinate 5 mM, and rotenone 2 μM. ADP was injected at 3.5 mM to induce state 3 from state 2, FCCP at 4 μM to measure maximal respiration, and antimycin A at 4 μM to measure non‐mitochondrial electron transport chain oxygen consumption and subtract it. Mix and measurements were performed as previously reported for the XF24 9.

### Genotyping and PCR

Genotyping was performed by PCR of tail snip lysates (Viagen, DirectPCR, Los Angeles, CA, USA) obtained during the weaning of pups. Floxed Mfn2 transgene was detected by PCR using GoTaq Green Master Mix (Promega, Madison, WI, USA) and the following primers were used: gaa gta ggc agt ctc cat cg and ccc aag aag agc atg tgt gc. The unexcised conditional band is 810 bp and the WT is 710 bp. Cre transgene was detected in the same samples by following the genotyping protocol provided by Jax laboratories using the following primers: gcg gtc tgg cag taa aaa cta tc and gtg aaa cag cat tgc tgt cac tt.

### Western blot

Protein extracts were subjected to SDS–polyacrylamide gel electrophoresis and immunoblotting using the following primary antibodies: Mfn2 (Abcam), Ucp1 (Abcam), and Tomm20 (Abcam), OXPHOS cocktail (Abcam).

### IHC/H&E staining

Slides of formalin‐fixed BAT containing 5‐μm paraffin‐embedded sections from Ucp1‐cre^(−/−)^.Mfn2^flox/flox^ and Ucp1‐cre^(+/−)^. Mfn2^flox/flox^ mice were deparaffinized, stained with Tomm20 by immunohistochemistry, and imaged as previously described 9. Sections (5 μm) were also stained with hematoxylin and eosin.

### Lipid droplet quantification

H&E slides were imaged and analyzed by Image J. Region of interest was created around the cell edges as well as all the lipid droplets per cell, and the area for each region of interest was measured.

### Statistical analysis

Student's *t*‐test, two‐way ANOVA, and ANCOVA were performed using Graph Pad prism and Excel as noted in the legends. *P*‐value < 0.05 was considered significant.

### RNA isolation and RT–PCR analysis

RNA was isolated following the manufacturer's protocol for the RNeasy Kit (QIAGEN). First‐strand cDNA synthesis from total RNA template was performed with Bio‐Rad IScript cDNA Synthesis System, followed by SYBR Green qPCR amplification. Normalization was performed using specific amplification of *Cyclophilin A,* and qPCRs were performed in triplicate for each biological experiment. Data are shown as sample mean between triplicate experiment ± standard deviation. Significance was calculated by paired Student's *t*‐test. Primer sequences used for each specific genes are available on request.

### Mitochondrial content

Total DNA was extracted from BAT using QuickExtract DNA Extraction Solution 1.0 (Epicenter) following the manufacturer's instructions. DNA amplification of the mitochondrial‐encoded NADH dehydrogenase 1 (mt‐ND1) relative to nuclear TFAM was used to determine mitochondrial DNA copy numbers.

### Electron microscopy

BAT were dissected and fixed in 0.5% glutaraldehyde and 4% paraformaldehyde (EM Grade) in 0.1 M phosphate buffer and kept at 4°C for 24 h. Tissues were then transferred to 0.1 M phosphate buffer (pH = 7.4) and sent for EM processing to Washington University School of Medicine Department of Otolaryngology.

### Cholesterol and triglyceride measurements

Serum samples were loaded in IDEXX Chemistry Analyzer for TG and Cholesterol measurement using colorimetric assay measurements.

## Author contributions

KM and ML wrote the paper, performed experiments, and designed the study. IYB contributed with electron microscopy analysis and with data interpretation. SS, RAG, LS, and TA performed mouse work and histology experiments. ER performed Western blots and genotyping. VE‐Z and KMT performed image analysis. MC and VP performed mtDNA quantification and mRNA measurements. MFO, VP, and BEC helped with experiments and data interpretation. OSS and ML designed and directed the study, and supervised manuscript writing.

## Conflict of interest

The authors declare that they have no conflict of interest.

## Supporting information



Appendix

Expanded View Figures PDF

Review Process File

## References

[1] Cannon B , Nedergaard J (2004) Brown adipose tissue: function and physiological significance. Physiol Rev 84: 277–359 14715917 10.1152/physrev.00015.2003

[2] Nicholls DG , Bernson VS , Heaton GM (1978) The identification of the component in the inner membrane of brown adipose tissue mitochondria responsible for regulating energy dissipation. Experientia Suppl 32: 89–93 348493 10.1007/978-3-0348-5559-4_9

[3] Nicholls DG (2001) A history of UCP1. Biochem Soc Trans 29: 751–755 11709069 10.1042/bst0290751

[4] Shimizu I , Aprahamian T , Kikuchi R , Shimizu A , Papanicolaou KN , MacLauchlan S , Maruyama S , Walsh K (2014) Vascular rarefaction mediates whitening of brown fat in obesity. J Clin Invest 124: 2099–2112 24713652 10.1172/JCI71643 PMC4001539

[5] Duteil D , Tosic M , Lausecker F , Nenseth HZ , Muller JM , Urban S , Willmann D , Petroll K , Messaddeq N , Arrigoni L *et al* (2016) Lsd1 ablation triggers metabolic reprogramming of brown adipose tissue. Cell Rep 17: 1008–1021 27760309 10.1016/j.celrep.2016.09.053 PMC5081406

[6] Liesa M , Palacin M , Zorzano A (2009) Mitochondrial dynamics in mammalian health and disease. Physiol Rev 89: 799–845 19584314 10.1152/physrev.00030.2008

[7] Liesa M , Shirihai OS (2013) Mitochondrial dynamics in the regulation of nutrient utilization and energy expenditure. Cell Metab 17: 491–506 23562075 10.1016/j.cmet.2013.03.002 PMC5967396

[8] Soriano FX , Liesa M , Bach D , Chan DC , Palacin M , Zorzano A (2006) Evidence for a mitochondrial regulatory pathway defined by peroxisome proliferator‐activated receptor‐gamma coactivator‐1 alpha, estrogen‐related receptor‐alpha, and mitofusin 2. Diabetes 55: 1783–1791 16731843 10.2337/db05-0509

[9] Wikstrom JD , Mahdaviani K , Liesa M , Sereda SB , Si Y , Las G , Twig G , Petrovic N , Zingaretti C , Graham A *et al* (2014) Hormone‐induced mitochondrial fission is utilized by brown adipocytes as an amplification pathway for energy expenditure. EMBO J 33: 418–436 24431221 10.1002/embj.201385014 PMC3983686

[10] Feldmann HM , Golozoubova V , Cannon B , Nedergaard J (2009) UCP1 ablation induces obesity and abolishes diet‐induced thermogenesis in mice exempt from thermal stress by living at thermoneutrality. Cell Metab 9: 203–209 19187776 10.1016/j.cmet.2008.12.014

[11] Liu X , Rossmeisl M , McClaine J , Riachi M , Harper ME , Kozak LP (2003) Paradoxical resistance to diet‐induced obesity in UCP1‐deficient mice. J Clin Invest 111: 399–407 12569166 10.1172/JCI15737 PMC151850

[12] Cui X , Nguyen NL , Zarebidaki E , Cao Q , Li F , Zha L , Bartness T , Shi H , Xue B (2016) Thermoneutrality decreases thermogenic program and promotes adiposity in high‐fat diet‐fed mice. Physiol Rep 4: e12799. 27230905 10.14814/phy2.12799 PMC4886167

[13] Guaras A , Perales‐Clemente E , Calvo E , Acin‐Perez R , Loureiro‐Lopez M , Pujol C , Martinez‐Carrascoso I , Nunez E , Garcia‐Marques F , Rodriguez‐Hernandez MA *et al* (2016) The CoQH2/CoQ ratio serves as a sensor of respiratory chain efficiency. Cell Rep 15: 197–209 27052170 10.1016/j.celrep.2016.03.009

[14] Chen H , McCaffery JM , Chan DC (2007) Mitochondrial fusion protects against neurodegeneration in the cerebellum. Cell 130: 548–562 17693261 10.1016/j.cell.2007.06.026

[15] Bartelt A , Bruns OT , Reimer R , Hohenberg H , Ittrich H , Peldschus K , Kaul MG , Tromsdorf UI , Weller H , Waurisch C *et al* (2011) Brown adipose tissue activity controls triglyceride clearance. Nat Med 17: 200–205 21258337 10.1038/nm.2297

[16] Prentki M , Matschinsky FM , Madiraju SR (2013) Metabolic signaling in fuel‐induced insulin secretion. Cell Metab 18: 162–185 23791483 10.1016/j.cmet.2013.05.018

[17] Koliaki C , Szendroedi J , Kaul K , Jelenik T , Nowotny P , Jankowiak F , Herder C , Carstensen M , Krausch M , Knoefel WT *et al* (2015) Adaptation of hepatic mitochondrial function in humans with non‐alcoholic fatty liver is lost in steatohepatitis. Cell Metab 21: 739–746 25955209 10.1016/j.cmet.2015.04.004

[18] Amati F , Dube JJ , Alvarez‐Carnero E , Edreira MM , Chomentowski P , Coen PM , Switzer GE , Bickel PE , Stefanovic‐Racic M , Toledo FG *et al* (2011) Skeletal muscle triglycerides, diacylglycerols, and ceramides in insulin resistance: another paradox in endurance‐trained athletes? Diabetes 60: 2588–2597 21873552 10.2337/db10-1221 PMC3178290

[19] Pich S , Bach D , Briones P , Liesa M , Camps M , Testar X , Palacin M , Zorzano A (2005) The Charcot‐Marie‐Tooth type 2A gene product, Mfn2, up‐regulates fuel oxidation through expression of OXPHOS system. Hum Mol Genet 14: 1405–1415 15829499 10.1093/hmg/ddi149

[20] Sebastian D , Hernandez‐Alvarez MI , Segales J , Sorianello E , Munoz JP , Sala D , Waget A , Liesa M , Paz JC , Gopalacharyulu P *et al* (2012) Mitofusin 2 (Mfn2) links mitochondrial and endoplasmic reticulum function with insulin signaling and is essential for normal glucose homeostasis. Proc Natl Acad Sci USA 109: 5523–5528 22427360 10.1073/pnas.1108220109 PMC3325712

[21] Cohen P , Levy JD , Zhang Y , Frontini A , Kolodin DP , Svensson KJ , Lo JC , Zeng X , Ye L , Khandekar MJ *et al* (2014) Ablation of PRDM16 and beige adipose causes metabolic dysfunction and a subcutaneous to visceral fat switch. Cell 156: 304–316 24439384 10.1016/j.cell.2013.12.021 PMC3922400

[22] Tan CY , Virtue S , Bidault G , Dale M , Hagen R , Griffin JL , Vidal‐Puig A (2015) Brown Adipose tissue thermogenic capacity is regulated by Elovl6. Cell Rep 13: 2039–2047 26628376 10.1016/j.celrep.2015.11.004 PMC4688035

[23] Boutant M , Kulkarni SS , Joffraud M , Ratajczak J , Valera‐Alberni M , Combe R , Zorzano A , Cantó C (2017) Mfn2 is critical for brown adipose tissue thermogenic function. EMBO J 36: 1543–1558 28348166 10.15252/embj.201694914 PMC5452040

