# Supplementary material for: Mfn2 deletion in brown adipose tissue protects from insulin resistance and impairs thermogenesis
Source: EMBO Rep. 2017 May 24;18(7):1123–38. doi: 10.15252/embr.201643827 (PMC5887905; doi:10.15252/embr.201643827)
Supplement: Supplementary file 1 — Appendix [file EMBR-18-1123-s001.pdf]

APPENDIX

Mfn2 deletion in brown adipose tissue protects from insulin resistance and impairs thermogenesis.

Kiana Mahdaviani, Ilan Benador, Shi Su, Raffi Gharakhanian, Linsey Stiles, Kyle Trudeau, Maria Cardamone, Violeta Enríquez, Eleni Ritou, Tamar Aprahamian, Marcus F. Oliveira, Barbara E. Corkey, Valentina Perissi, Marc Liesa and Orian S. Shirihai

Table of Contents

Appendix Figure Legends S1-S5 pages 2-4

Appendix Figures S1-S5 pages 5-9

APPENDIX FIGURE LEGENDS

Appendix Figure S1. BAT-Mfn2-KO females do not show changes in food intake, activity or body composition measured by NMR, despite resistance to obesity.

A) Quantification of body composition measurements on n=3-4 control (WT) and BAT-Mfn2-KO female mice at 8 months old under chow diet. Data are presented as % fat and % lean normalized to corresponding mice total body weights.

B and C) Quantification of food intake calculated as gram of food per mouse per week in wild type (WT) and BAT-Mfn2-KO female mice fed a HFD at 22 °C (B) or at 30°C (C) respectively. Student t-test, unpaired $p>0.05$.

D) Quantification of locomotor activity measured as total x/y/z laser break counts over a 24 hour period in control (WT) and BAT-Mfn2-KO female mice fed a HFD at 30°C. Bars represent average of n=4-6 mice per genotype ± SEM. Student t-test, unpaired WT vs KO $p=0.17$.

Appendix Figure S2. Loss of Mfn2 in brown adipose tissue does not affect mitochondrial mass, but causes fragmentation.

A) Representative TEM images of tissue sections of BAT isolated from control (WT) and BAT-Mfn2-KO (KO) female mice fed a chow diet.

B and C) Quantification of mitochondrial morphology by Form factor (B) and aspect ratio (C) of each individual mitochondrion from BAT sections obtained from n=3 control and BAT-Mfn2-KO female mice per group. (*) represents Student t-test, unpaired WT vs KO $p< 0.05$.

D) Representative TEM images of tissue sections of BAT isolated from control (WT) and BAT-Mfn2-KO (KO) female mice fed a chow diet at a lower magnification.

E and F) Quantification of mitochondrial mass by mitochondrial number per cell (E) and mitochondrial area (F) of each individual mitochondrion from BAT sections obtained from n=3 control and BAT-Mfn2-KO female mice per group. Student t-test, unpaired $p< 0.05$.

G) Representative images of IHC tissue sections of BAT isolated from control (WT) and BAT-Mfn2-KO (KO) male mice. Mitochondria are visualized by Tomm20 staining.

H) Protein levels quantification of Tomm20 per microgram of protein loaded, from BAT lysate isolated from n=3-5 control and BAT-Mfn2-KO female mice per group. Student t-test, unpaired $p< 0.05$.

I) Quantification of mitochondrial mass by mitochondrial DNA normalized by nuclear DNA on BAT extracted DNA isolated from n=3 control and BAT-Mfn2-KO female mice per group. (*) represents Student t-test, unpaired WT vs KO $p< 0.05$.

Appendix Figure S3. No differences in coupling of BAT mitochondria isolated from BAT-Mfn2-KO and wild type mice fed a chow diet or a HFD, when respiration is driven by complex I or II.

A and B) Quantification of the respiratory control ratio (RCR, state3/state2) measured in isolated BAT mitochondria from n=4-5 control (WT) and BAT-Mfn2-KO female (A) and n=13-19 male (B) mice per group fed a chow diet using different fuels. Bar graphs represent average ± SEM of RCR values. Unpaired Student t-test, $p<0.05$.

C) Quantification of the respiratory control ratio (RCR, state3/state2) measured in isolated BAT mitochondria from n=3-6 control (WT) and BAT-Mfn2-KO male mice per group fed a HFD at 22°C. Bar graphs represent average ± SEM for complex I driven-respiration (pyruvate- malate) and complex II-driven respiration (succinate-rotenone). Unpaired Student t-test, $p<0.05$. These low RCR are characteristic of BAT mitochondria in the presence of GDP and 0.1 % BSA, as previously described (Christiansen et al., 1973;Pedersen, 1970).

Appendix Figure S4. Loss of Mfn2 in brown adipose tissue under diet-induced obesity increases mitochondrial swelling and circularity, without changing cristae density, but reduces mitochondrial DNA levels.

A) Representative TEM images of tissue sections of BAT isolated from control (WT) and BAT-Mfn2-KO (KO) female mice fed a HFD at 22°C.

B and C) Quantification of mitochondrial morphology by Form factor (B) and aspect ratio (C) values of each individual mitochondrion from BAT sections obtained from n=2-3 control and BAT-Mfn2-KO female mice per group. (*) represents Student t-test, unpaired WT vs KO $p< 0.05$.

D and E) Quantification of mitochondrial area and cristae values of each individual mitochondrion from BAT sections obtained from n=2-3 control and BAT-Mfn2-KO female mice per group. (*) represents Student t-test, unpaired WT vs KO $p < 0.05$.

F) Quantification of mitochondrial mass by mitochondrial DNA normalized by nuclear DNA on BAT extracted DNA isolated from n=2-3 control and BAT-Mfn2-KO female mice fed a HFD at 22°C or 30°C per group. (*) represents Student t-test, unpaired WT vs KO $p < 0.05$.

Appendix Figure S5. BAT-Mfn2-KO obese females do not show significant enhancement of beiging in scWAT and do not show increased plasma levels of FGF21.

A) Quantification of RT-PCR analysis for Ucp1 gene expression in WAT-Sc of control (WT) and BAT-Mfn2-KO female mice fed a high fat diet (HFD) at either 22°C or 30°C. Ucp1 gene expression values are normalized against CypA gene expression. Data is represented as fold change over the control (WT) group at HFD 30°C. (n=2-5 per group). Bar graphs represent average ± SEM. * represents Student t-test, unpaired HFD 22°C vs. HFD 30°C $p<0.05$.

B) Representative Western blot measuring Creatine Kinase (CK), Tomm20 and GAPDH on WAT-Sc total lysates from control (WT) and BAT-Mfn2-KO female mice fed a high fat diet (HFD) at either 22°C or 30°C.

C and D) Protein level quantification of CK protein levels normalized to their corresponding loading control (GAPDH). Bars represent average of CK/GAPDH from n=4-6 female mice at HFD 22°C (C) or at HFD 30°C (D) per group ±SEM.

E and F) Protein level quantification of Tomm20 protein levels normalized to their corresponding loading control (GAPDH). Bars represent average of Tomm20/GAPDH from n=4-6 female mice at HFD 22°C (C) or at HFD 30°C (D) per group ±SEM.

G) Quantification of serum FGF21 levels of control (WT) and BAT-Mfn2-KO female mice fed a high fat diet at 22°C (n=7-11 per group) or 30°C (n=5 per group). Bars represent average of serum FGF21 levels (pg/mL) ±SEM.

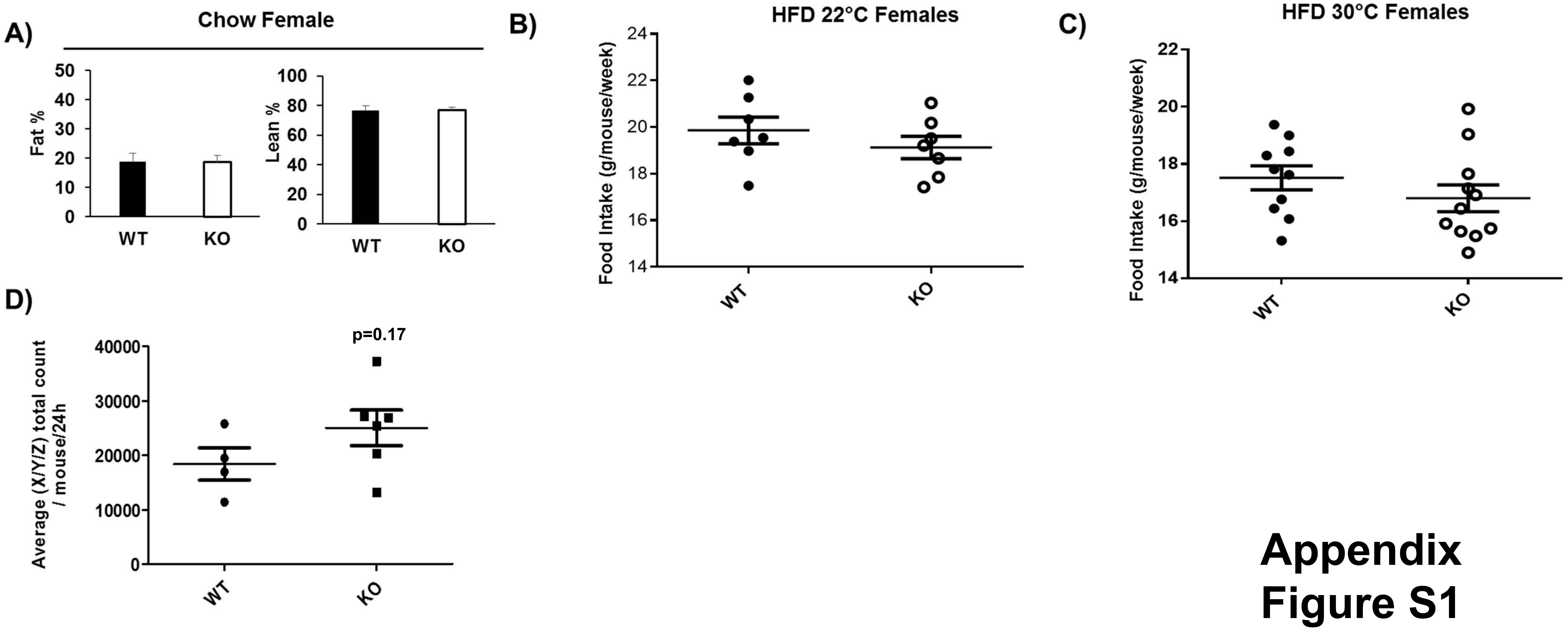

Appendix Figure S1

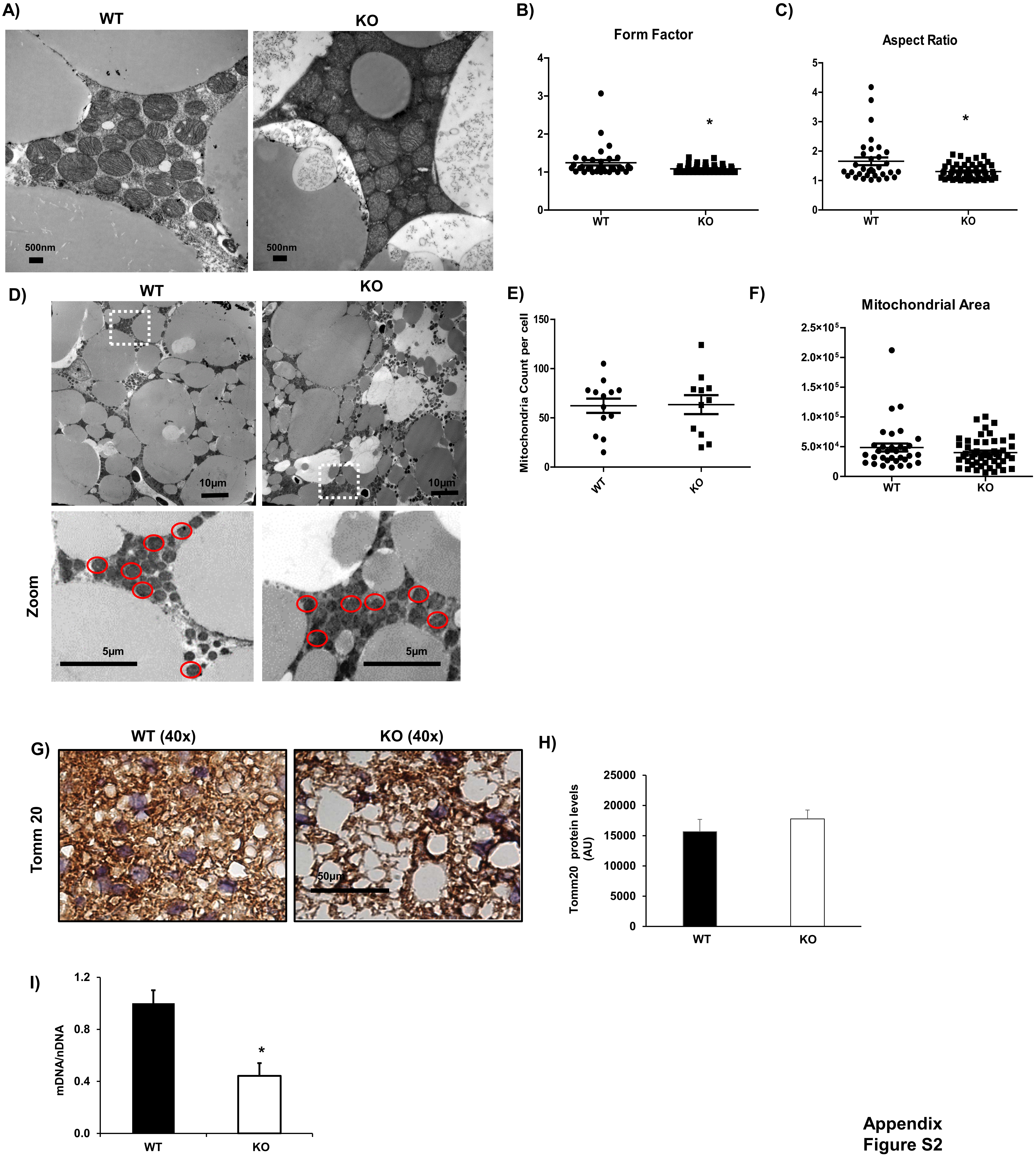

Appendix Figure S2

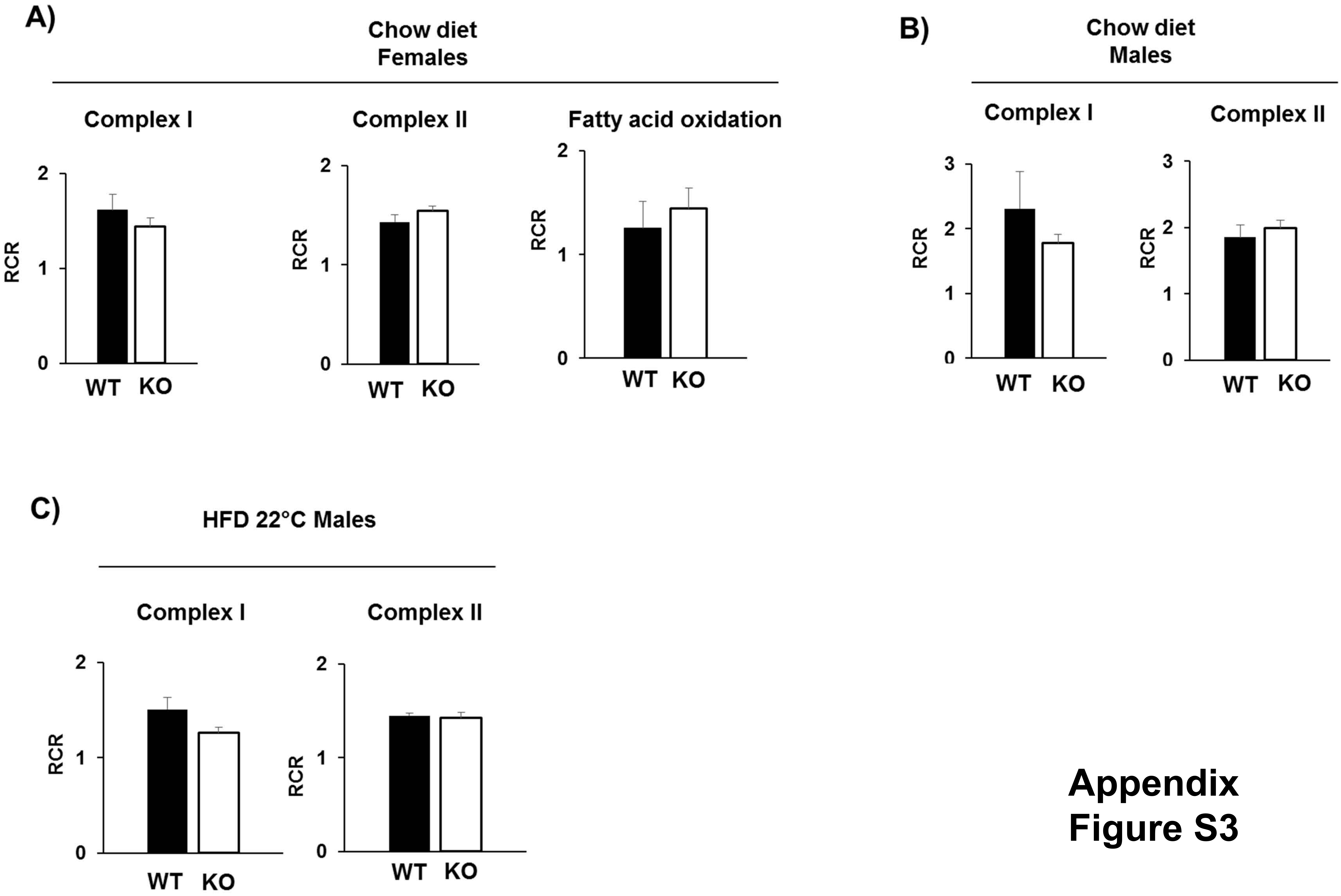

Appendix Figure S3

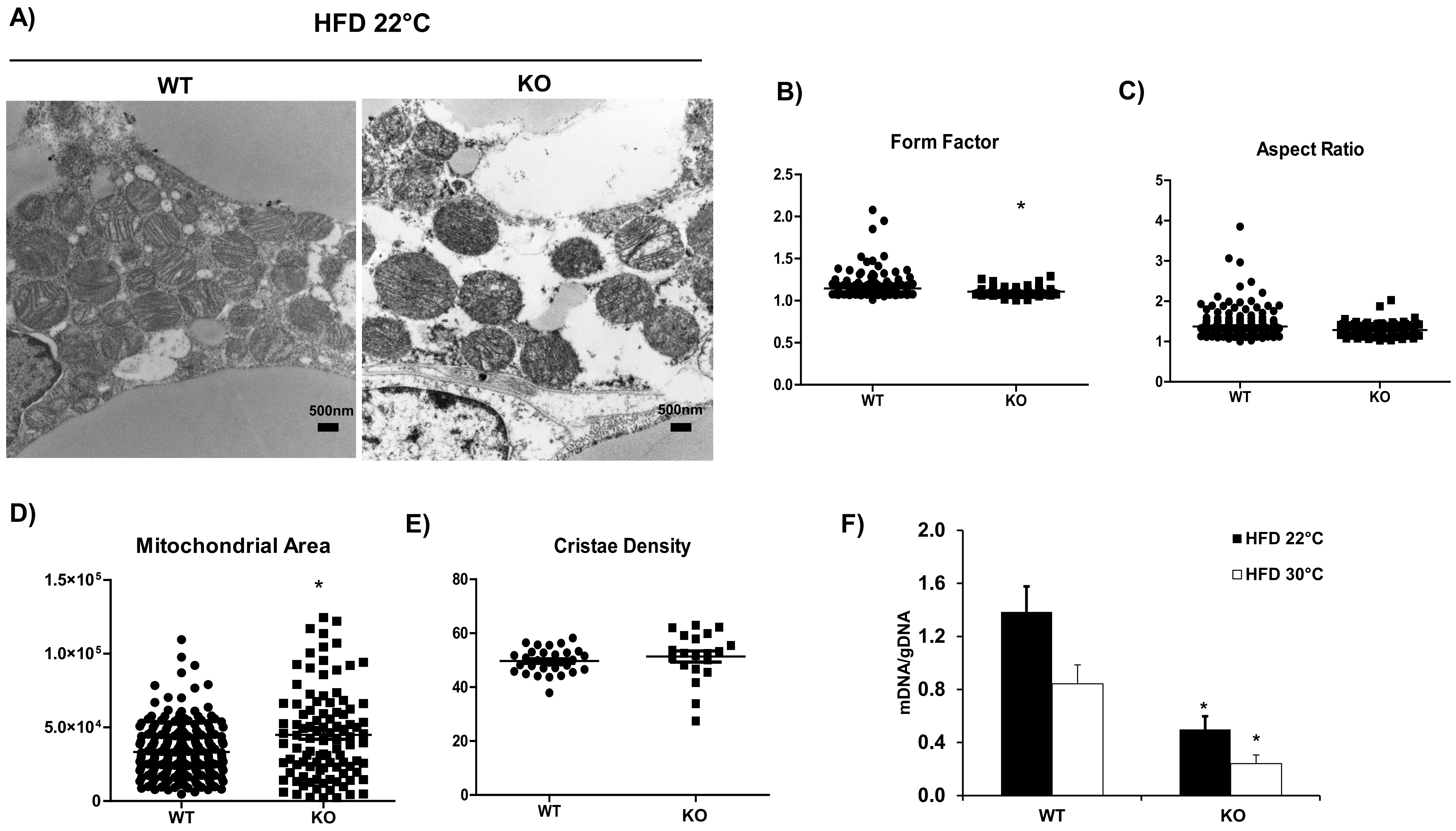

Appendix Figure S4

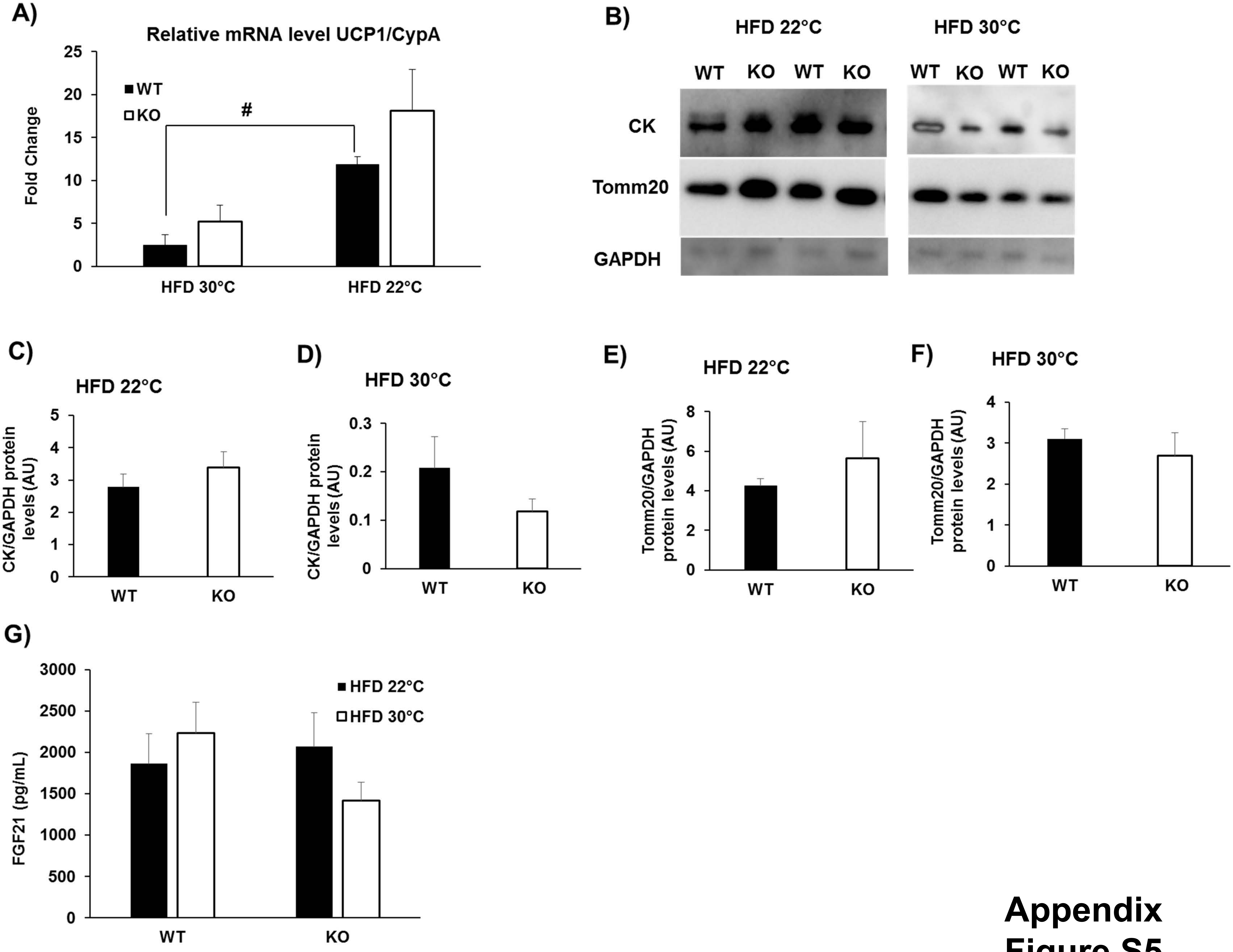

Appendix Figure S5
