# Supplementary material for: Mfn2 deletion in brown adipose tissue protects from insulin resistance and impairs thermogenesis
Source: EMBO Rep. 2017 May 24;18(7):1123–38. doi: 10.15252/embr.201643827 (PMC5887905; doi:10.15252/embr.201643827)
Supplement: Supplementary file 2 — Expanded View Figures PDF [file EMBR-18-1123-s002.pdf]

Expanded View Figures

Figure EV1. Diet-induced obesity increases Mfn2 and mitochondrial respiratory capacity in BAT.

A Representative Western blot measuring Mfn2, Tomm20, and Porin on BAT total lysates from wild type female mice fed a chow diet or a high fat diet (HFD).

B Protein level quantification of Tomm20 and Porin per microgram of protein loaded, as well as Mfn2 protein levels normalized to their corresponding loading control (Tomm20). Bars represent average of Tomm20, Porin, and Mfn2/Tomm20 from n = 3–8 female mice per group ± SEM.

C Quantification of mitochondrial DNA (mtDNA) normalized to nuclear DNA (nDNA) by qPCR on extracted DNA from BAT of wild type female mice fed a chow diet or a HFD at 22°C or 30°C. Bars represent average of mDNA/nDNA from n = 3 female mice per group ± SEM.

D Representative EM images of BAT extracted from wild type female mice fed a chow diet or a high fat diet (HFD).

E Representative Western blot measuring complex I subunit Ndufb8, complex II Sdhb, complex III Uqcrc2, complex IV Cox1, and outer mitochondrial membrane Tomm20 in BAT total lysates from wild type female mice fed a chow or HFD.

F Protein level quantification of the four complex subunits normalized by corresponding Tomm20 levels. Bar graphs represent average ± SEM of complexes normalized to Tomm20 from n = 5–8 female mice per group fed a chow diet or a HFD.

G Representative Western blot measuring uncoupling protein 1 (Ucp1) and outer mitochondrial membrane Porin in BAT total lysates from wild type female mice fed a chow or HFD.

H Protein level quantification of Ucp1 and the corresponding loading control Porin levels. Bar graphs represent average ± SEM of Ucp1 normalized to Porin from n = 5–8 wild type female mice per group fed a chow diet or a HFD.

I–K Quantification of oxygen consumption rates (OCR) in BAT isolated mitochondria from wild type female mice fed a chow diet or a HFD under the different respiratory states. State 2 quantifies respiration driven by proton leak (no-ATP synthesis), state 3 quantifies respiration linked to maximal ATP synthesis, and maximal represents maximal electron transport chain activity induced by FCCP. Bar graphs represent average ± SEM for complex I-driven respiration (pyruvate + malate, n = 4–8 mice per group) (I), complex II-driven respiration (succinate—rotenone, n = 4–8 mice per group) (J), and fatty acid oxidation (palmitoyl carnitine—malate, n = 3–6 mice per group) (K).

L–N Quantification of OCR in BAT isolated mitochondria from wild type male mice fed a chow diet or a HFD under the different respiratory states. State 2 quantifies respiration driven by proton leak (no-ATP synthesis), state 3 quantifies respiration linked to maximal ATP synthesis, and maximal represents maximal electron transport chain activity induced by FCCP. Bar graphs represent average ± SEM for complex I-driven respiration (pyruvate + malate, n = 3–13 mice per group) (L), complex II-driven respiration (succinate—rotenone, n = 3–13 mice per group) (M), and fatty acid oxidation (palmitoyl carnitine-malate, n = 2 mice per group).

O Mouse body temperature measurements from n = 5–11 wild type female mice per group at 9 months old and fed a chow diet, a high fat diet (HFD) either at 22°C or at thermoneutrality 30°C. Values shown are means ± SEM.

Data information: Statistical analysis: * represents significance using Student's t-test, unpaired $P < 0.05$.

© 2017 The Authors

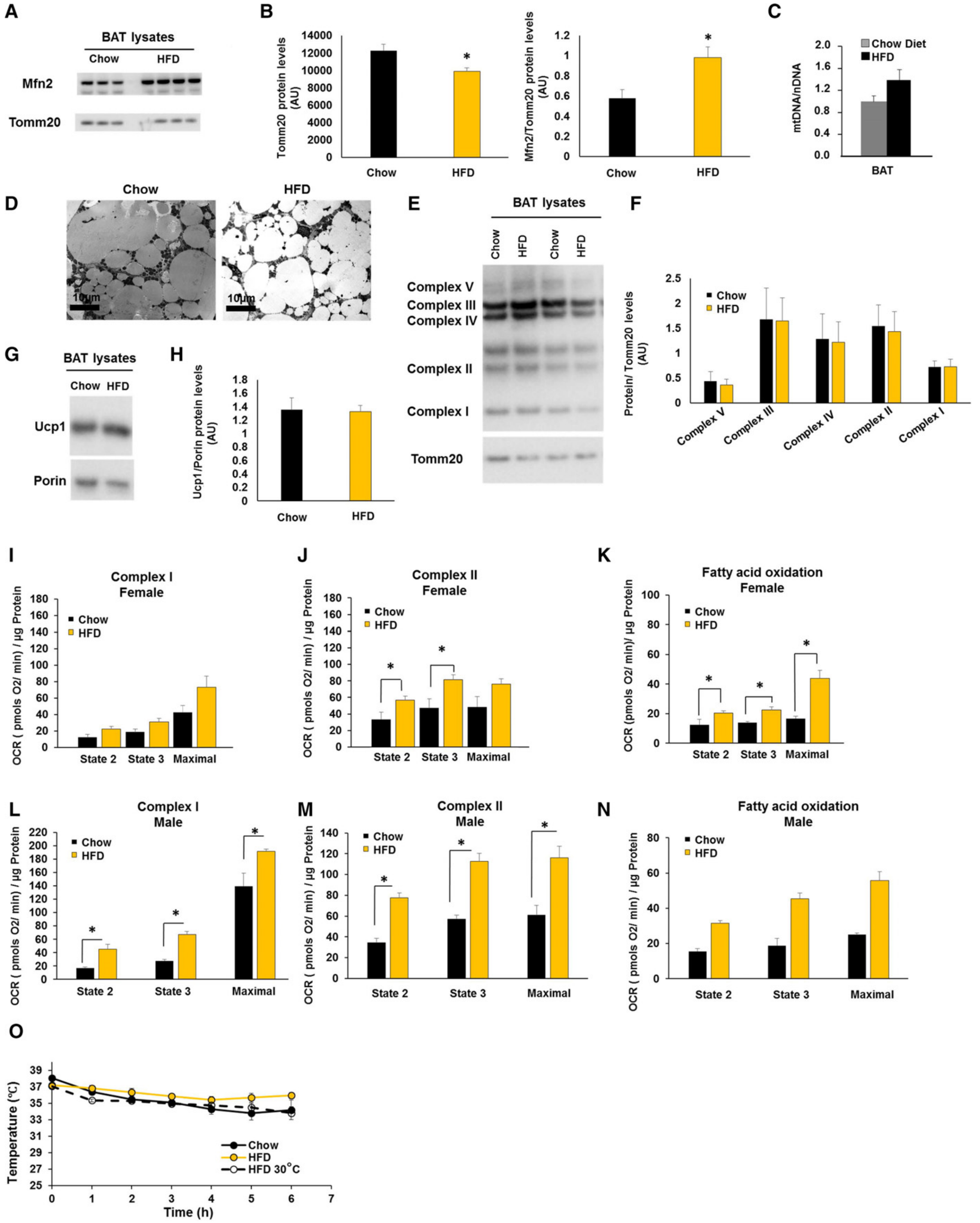

Figure EV1.

 © 2017 The Authors

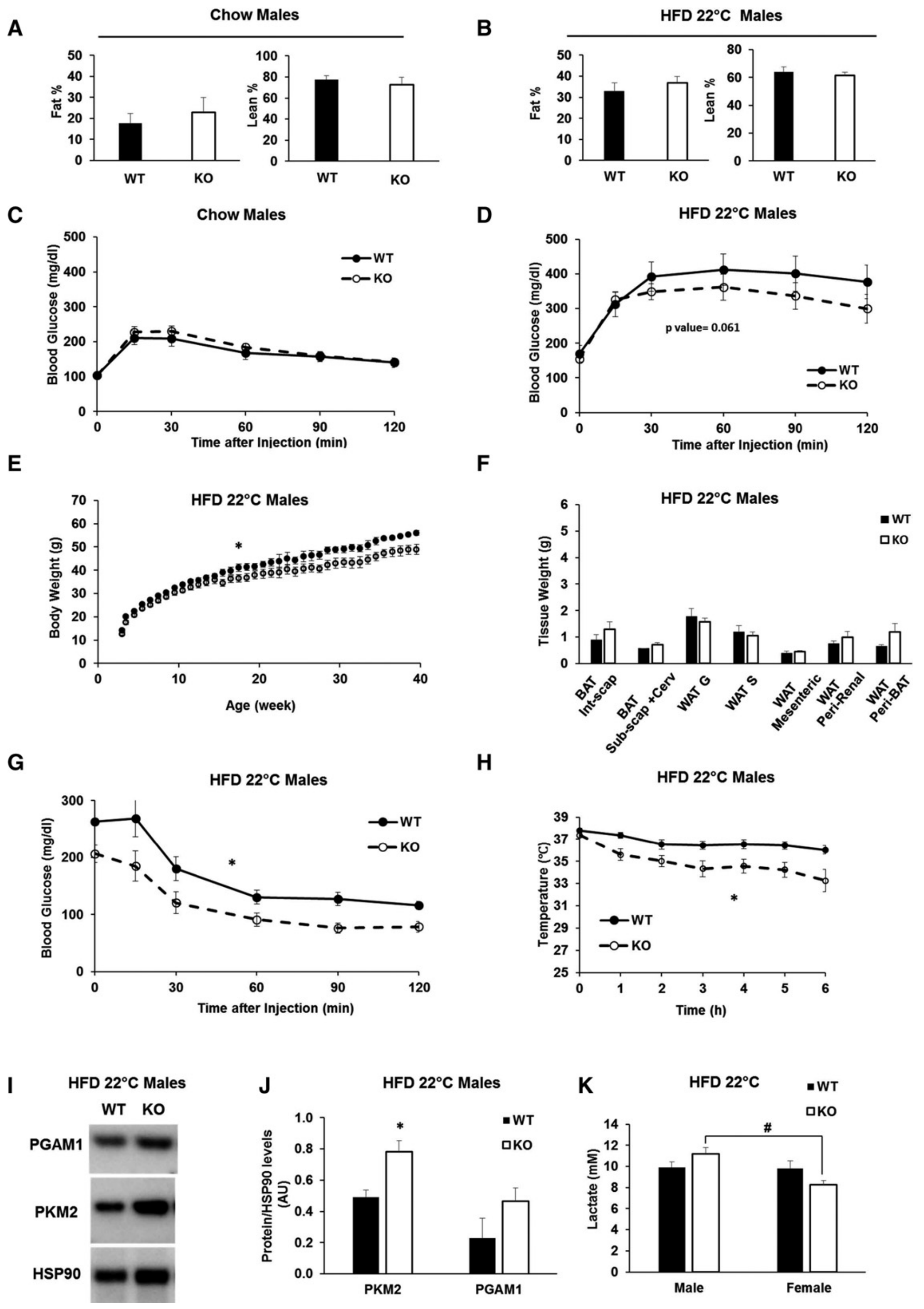
A
Chow Males
Fat %
Lean %
WT
KO
B
HFD 22°C Males
C
Chow Males
Blood Glucose (mg/dl)
Time after Injection (min)
D
HFD 22°C Males
p value= 0.061
E
HFD 22°C Males
Body Weight (g)
Age (week)
F
HFD 22°C Males
Tissue Weight (g)
BAT Int-scap
BAT Sub-scap +Cerv
WAT G
WAT S
WAT Mesenteric
WAT Peri-Renal
WAT Peri-BAT
G
HFD 22°C Males
H
HFD 22°C Males
Temperature (°C)
Time (h)
I
HFD 22°C Males
PGAM1
PKM2
HSP90
J
HFD 22°C Males
Protein/HSP90 levels (AU)
K
HFD 22°C
Lactate (mM)
Male
Female

Figure EV2.

Figure EV2. BAT-Mfn2-KO male mice are protected from HFD-induced insulin resistance and increase BAT glycolytic capacity, despite showing the same body fat gain and being cold-intolerant.

A Body composition of n = 8–9 wild type (WT) and BAT-Mfn2-KO (KO) male mice (7 months) per group on chow diet. Bar graph represent average of % fat and % lean mass of total body weight ± SEM.
B Body composition of n = 4–6 wild type (WT) and BAT-Mfn2-KO (KO) male mice (7 months) per group on a HFD at ambient temperature (22°C). Bar graph represent average of % fat and % lean mass of total body weight ± SEM.
C Glucose tolerance test (GTT) on n = 11–16 wild type (WT) and BAT-Mfn2-KO (KO) male mice per group at 5 months old, fed chow diet. Values shown are average ± SEM.
D GTT on n = 10–13 wild type (WT) and BAT-Mfn2-KO (KO) male mice per group, fed a HFD at 22°C. Values shown are average ± SEM. Two-way ANOVA test, WT vs. KO, $P < 0.05$.
E Body weight measurements of n = 8–9 wild type (WT) and BAT-Mfn2-KO (KO) male mice per group on HFD at an ambient temperature of 22°C (room temperature, RT) over 40 weeks. Values shown are average ± SEM. Two-way ANOVA test, WT vs. KO, *$P < 0.05$.
F Quantification of WAT and BAT deposit weight isolated from n = 4–6 wild type (WT) and BAT-Mfn2-KO (KO) male mice per group fed a HFD at ambient temperature (22°C). Bar graphs represent average ± SEM. * represents significance using Student's t-test, unpaired $P < 0.05$.
G Insulin tolerance tests (ITT) on n = 9–13 wild type (WT) and BAT-Mfn2-KO (KO) male mice per group, fed a HFD at ambient temperature (22°C). Values shown are average ± SEM. Two-way ANOVA test, WT vs. KO, *$P < 0.05$.
H Body temperature measurements of wild type (WT) and BAT-Mfn2-KO (KO) male (n = 10–12) mice at 9 months old in HFD groups at ambient temperature. Values shown in both panels are means ± SEM. * represents significance using two-way ANOVA test, WT vs. KO, $P < 0.05$.
I Representative Western blot measuring PGAM1, PKM2, and HSP90 in BAT total lysates from wild type (WT) and BAT-Mfn2 KO (KO) males on HFD.
J Protein level quantification of PGAM1 and PKM2 normalized by HSP90 level, used as loading control. Bar graphs represent average ± SEM of proteins normalized to HSP90 from n = 3–4 mice per group of wild type (WT) and BAT-Mfn2-KO (KO) male mice. * represents significance using Student's t-test, unpaired $P < 0.05$.
K Quantification of serum lactate from n = 3–6 male and n = 4–8 female mice per group of wild type (WT) and BAT-Mfn2-KO (KO) mice. Bar graphs represent average ± SEM of serum lactate (mM). $^{\#}$ represents significance between male KO and female KO groups using Student's t-test, unpaired $P < 0.05$.

© 2017 The Authors
