# Supplementary material for: Mfn2 deletion in brown adipose tissue protects from insulin resistance and impairs thermogenesis
Source: EMBO Rep. 2017 May 24;18(7):1123–38. doi: 10.15252/embr.201643827 (PMC5887905; doi:10.15252/embr.201643827)
Supplement: Supplementary file 3 — Review Process File [file EMBR-18-1123-s003.pdf]

EMBO
*reports*

Manuscript EMBO-2016-43827

# Mfn2 deletion in brown adipose tissue protects from insulin resistance and impairs thermogenesis

Kiana Mahdaviani, Ilan Benador, Shi Su, Raffi Gharakhanian, Linsey Stiles, Kyle Trudeau, Maria Cardamone,Violeta Enriquez, Eleni Ritou, Tamar Aprahamian, Marcus F. Oliveira, Barbara E. Corkey, Valentina Perissi, Marc Liesa, and Orian S. Shirihai

*Corresponding authors: Marc Liesa (Boston University School of Medicine, David Geffen School of Medicine at UCLA); Orian S. Shirihai (Boston University School of Medicine, David Geffen School of Medicine at UCLA; Ben Gurion University School of Medicine)*

**Review timeline:**

| | |
|---|---|
| Transfer date: | 16 December 2016 |
| Editorial Decision: | 13 January 2017 |
| Appeal received: | 18 January 2017 |
| Editorial Decision: | 30 January 2017 |
| Revision received: | 15 March 2017 |
| Editorial Decision: | 30 March 2017 |
| Revision received: | 03 April 2017 |
| Accepted: | 10 April 2017 |

Editor: Martina Rembold

**Transaction Report: Please note that this manuscript was transferred from *The EMBO Journal,* where it was originally reviewed. The outstanding referee comments from that journal at the time of transfer are included in the first editorial decision letter shown here.**

(Note: With the exception of the correction of typographical or spelling errors that could be a source of ambiguity, letters and reports are not edited. The original formatting of letters and referee reports may not be reflected in this compilation.)

1st Editorial Decision 13 January 2017

Thank you again for the submission of your manuscript to EMBO reports and for your patience, while it was seen by an editorial advisor, who is an expert in the field. I apologize for the delay in getting back to you, which is due to the Christmas holidays during which it is more difficult to get hold of people. The advisor evaluated your manuscript for potential publication in EMBO reports and I am sorry to say that the outcome of this evaluation is not a positive one.

In general the advisor pointed out that the data are in part conflicting, that important experiments are missing and that some of the observed effects are minor, yet these effects are central for the study's conclusions. More specifically, the advisor agreed with referee 1 that oxygen consumption rates are missing for Figure 1 and 3 and indicated that data on physical activity under RT and cold exposure needed to be added to fully describe the phenotype. The reported increase in FA oxidation capacity appears to contradict the lipohypertrophy in BAT from animals, in the opinion of the advisor. Moreover, the changes in UCP1 expression, the improvement in glucose tolerance as well as insulin sensitivity seem minor. Finally, the advisor agreed with referee 1 that ..."an acute adrenergic challenge and then recording oxygen consumption is required to make solid claims." The advisor

© European Molecular Biology Organization 

concluded that "... while an observation that thermogenic capacity and improvements in energy homeostasis could be independent from each other may be interesting, I feel that the current manuscript falls short in providing convincing evidence in its current form."

Given this opinion and the opinion of the two referees who had evaluated the study for EMBO Journal and were also not supportive of publication, I have no other choice but to return your manuscript to you as we cannot offer to publish it. I am truly sorry to disappoint you on this occasion and wish you success with publication of your manuscript elsewhere.

---------------------

REFEREE REPORT FROM EMBO JOURNAL

**Referee #1:**

In this revised version, the authors have responded to the concerns raised in the initial review and provided additional information and experimental data. The two major experiments that were suggested: metabolic cages for direct BAT respiration and reproduce the mitochondrial data, were not provided. The authors interpret the defects in thermogenesis in response to acute cold as an "uncoupling defect" and suggest that under baseline (adapted) conditions there is no defect in BAT. This conclusion would have benefited from additional experiments suggested. This is a choice of the authors and these issues notwithstanding, there are no further experimental comments.

One area that the paper did not benefit from the revisions is the clarity and organization of the paper. The manuscript is extraordinarily difficult to read and understand. The organization of large amount of data can be much improved to make this a clearer manuscript. The same comments were made by the second reviewer and these should be considered by the authors. It was extremely difficult to go through this manuscript.

-Perhaps one way to achieve that would involve moving a substantial amount of data to the supplement (for example all of the male data) and combine some figures to reduce the number of main figures to 6 or 7. Perhaps the authors can describe the phenotypes of males and females together (as they are quite similar) and organize separate sections for mechanisms between maleas and females (basically the mitochondria respiration and RER). Figure 7 and perhaps 10 may be moved to supplement as well as some panels from crowded figures and combine some figures. (for example 5 and 11, and parts of 8 (A-C) with 4). These are some suggestions to consider for the authors.

Finally, the abstract may benefit from more clarity and the introduction may benefit from being shortened (for example paragraphs 2 and 3 may be significantly reduced or removed).

Appeal - authors' response 18 January 2017

We are providing here a point-by-point response to the comments from the advisor. The main concerns can be summarized in 3 points:

1) The observed effects are minor:

*While we agree that the significant improvements in glucose tolerance and insulin sensitivity at 22oC in BAT-Mfn2-KO mice are not as large as changes in their cold tolerance, these improvements in glucose metabolism are markedly amplified at thermoneutrality. This amplification demonstrates that Mfn2 deletion in brown adipose tissue (BAT) results in improved capacity to adapt to obesity without the need for a proper response to thermal stress. We thus suggest that deletion of Mfn2 unravel thermal stress-independent pathways of BAT that protect from glucose intolerance. The therapeutic relevance of such pathways is the treatment of insulin resistance in humans, without the need to expose them to cold and as such, the thermoneutrality findings are of greater relevance. Furthermore, in the manuscript that was submitted back to back with ours, data from a similar mouse strain had even milder effect on glucose tolerance and this was not a cause of concern for the same two reviewers that reviewed our manuscript in EMBO in the back-to-back*

*submission.*

*Regarding the mild effects on Ucp1 expression (34% reduction in chow diet), we do not conclude that this reduction is the only factor causing cold intolerance under chow diet. Indeed, we think that this reduction demonstrates a metabolic remodeling of BAT mitochondria to enhance coupled (not uncoupled) fatty acid oxidation, required to cover increased ATP demand induced by lipohypertrophy. In agreement with our conclusion, we observed that after diet-induced obesity and thermoneutrality, the differences in Ucp1 expression are reduced, but cold intolerance is maintained. Therefore, the magnitude of the decrease in Ucp1 does not change the fact that these mice are cold intolerant or any of the conclusions of the paper.*

2) Lipohypertrophy is contradictory to increased mitochondrial respiratory capacity:

*While the statement that lipid accumulation represents decreased energy expenditure is commonly raised to explain obesity, we know this is incorrect. Obesity leads to increased mitochondrial lipid utilization and the storage of lipids does not represent a state of impaired energy expenditure. Storage of lipid is energetically costly. Lipid droplets are dynamic and ATP-demanding lipid cycling is actively occurring in cells that accumulate lipid droplets. A first example is the pancreatic beta cells in obese animals. Beta cells that accumulate lipid droplets have increased energy expenditure due to lipid cycling, where lipid cycling is thought to play a role in preventing the detrimental effects of excess lipids (Prentki et al, Cell Met 2013). A second example is fatty liver where lipid accumulation is associated with increased lipid utilization and mitochondrial respiration (Koliaki et al, Cell Met 2015). A third example is athlete muscles, which show a marked increase in mitochondrial respiratory capacity, have been shown to accumulate lipid droplets (Amati et al, Diabetes 2011). In this regard, we show that the increased respiratory capacity caused by Mfn2 deletion will support the extra ATP production required to support lipohypertrophy. This conclusion is further supported by the two-fold increase in the central components of the lipid uptake machinery in BAT (LPL and CD36) at thermoneutrality. Upregulation of lipid uptake capacity is unlikely to represent a response to impaired lipid oxidation. Furthermore, previous studies show that severe BAT dysfunction, as any other tissue dysfunction, causes atrophy (i.e. such as abnormal Ucp1 overexpression) rather than energetically costly lipohypertrophy.*

3) Important experiments are missing:

*The advisor outlined the following: "advisor agreed with referee 1 that oxygen consumption rates are missing for Figure 1 and 3 and indicated that data on physical activity under RT and cold exposure needed to be added to fully describe the phenotype." We will be happy to perform the adrenergic stimulation-induced oxygen consumption experiments in mice if they are sufficient for the advisor to recommend publication and we will also be happy to provide physical activity data.*

*Beta adrenergic-stimulated oxygen consumption in mice will determine if impaired thermogenesis is a result of impaired adrenergic response or intrinsic to the adipocytes. However, the main hypothesis of our study is that BAT-mediated improvement in insulin sensitivity can be induced through a thermogenesis-independent pathway. As such, whichever results will come from experiments studying oxygen consumption in mice in response to beta 3-adrenergic agonists, they will not address the hypothesis tested in this study and, as such, they will not change any of the conclusions of the paper. In addition, it does not change our main conclusion that components of mitochondrial dynamics in BAT, such as Mfn2, can determine the metabolic response to diet induced obesity. In this case, we show that BAT-specific-fragmentation can protect from insulin resistance in the obese at thermoneutrality and this conclusion will not be changed by mice respiration experiments.*

*Thank you again for this opportunity to address the concerns from the advisor.*

2nd Editorial Decision — 30 January 2017

Thank you for your mail and the point-by-point response to the advisor's concerns.

I have meanwhile contacted both our advisor and referee #1 to inquire whether they would find your manuscript suitable for publication in EMBO reports if you were to provide the requested and

missing experiments. The advisor confirmed that the study is potentially suitable for publication in EMBO reports if ..."[the authors] provide the oxygen consumption experiments as well as the physical activity data for Figures 1 and 3 and the findings would be supportive...". Also referee 1 indicated that "... with those experiments in place, the paper will be suitable for publication in EMBO reports."

Given these positive opinions, I would like to invite you to revise the manuscript according to the referee's/advisor's suggestions. I would appreciate if you could send me a short note as to how much time you think you will need for these experiments. Also don't hesitate to contact me if you have any further questions or want to discuss the revision further.

I look forward to seeing a revised form of your manuscript when it is ready.

2nd Revision - authors' response 15 March 2017

Thank you for the opportunity to re-submit the manuscript. As requested by reviewer #1 and the advisor, we now provide whole body oxygen consumption measurements, as well as physical activity data. We would also like to thank the EMBOJ reviewer #1 and the advisor for this second opportunity, as their comments helped us to significantly improve the manuscript. In this letter, you will find a summary of the new data, which addresses the 2 major concerns raised by the reviewers:

1) New Figure 2I shows that whole body oxygen consumption rates after  3-agonist injection are the same in WT and BAT-Mfn2-KO anesthetized mice, despite their cold intolerance. These results demonstrate that: 1) the ability to stimulate brown adipose tissue lipolysis and oxygen consumption through  -3-receptors is intact in BAT-Mfn2-KO mice. 2) Whole body oxygen consumption coupled to ATP synthesis is replacing thermogenic Ucp1-dependent oxygen consumption in vivo. This is in agreement with our results showing that BAT-Mfn2-KO mice under chow diet have decreased Ucp1 levels and cold intolerance. Therefore, these data further support the results obtained in isolated mitochondria from BAT ex-vivo, which demonstrated absence of mitochondrial dysfunction and increased capacity for mitochondrial oxygen consumption linked to ATP synthesis in Mfn2-deleted mitochondria.

2) In new Supplementary Figure S2D, we provide physical activity data for BAT-Mfn2-KO mice fed a HFD under thermoneutrality (mice from Figure 3), as they show the largest degree of cold intolerance and the largest improvement in glucose tolerance. We do not detect differences in physical activity in the three axes measured between WT and BAT-Mfn2-KO mice (Figure S2D). These results demonstrate that changes in physical activity are neither driving nor contributing to the phenotypes caused by BAT-specific deletion of Mfn2 in obese mice at thermoneutrality.

While we are aware that we could not perform these measurements in all the experimental groups presented in this study, we are confident that the results shown are sufficient to confirm the major claims of our paper: that deletion of Mfn2 in BAT provides resistance to diet-induced glucose intolerance, increases BAT coupled mitochondrial respiratory capacity, BAT lipohypertrophy and cold-intolerance. However, despite not being essential for our major conclusions, as the advisor point of measuring physical activity in the rest of groups presented in our study is relevant, we have included the following paragraph in the discussion:

“As Mfn2 is primarily deleted in BAT of BAT-Mfn2-KO mice, their cold intolerance is unequivocally caused by a BAT-specific manipulation and, consequently, it can be expected to be exclusively a result of BAT mitochondrial remodeling. Exclusivity is strongly supported by the absence of changes in heat generation and physical activity in obese BAT-Mfn2-KO mice under thermoneutrality, the conditions in which BAT pathways related to cold exposure are shut down. Consequently, primary defects in other tissues would largely contribute to heat generation and physical activity at thermoneutrality and therefore the results we obtained exclude this possibility. However, we cannot exclude the existence of an alternative mechanism by which deletion of Mfn2 in BAT promotes the secretion of inhibitory factors leading to decreased shivering and/or thermogenic function in muscle, only apparent during acute cold exposure. We think that these specific questions describing elusive molecular mediators responsible for a potential crosstalk between BAT and muscle during acute cold exposure are beyond this study, but we can conclude

© European Molecular Biology Organization 

that BAT-Mfn2-KO mice can be used as a model to test these pathways.”

Finally, we have also added a second new paragraph in the discussion citing previous studies, from independent laboratories, which demonstrate that increased lipid accumulation in different tissues is associated with increased mitochondrial oxidative capacity coupled to ATP synthesis. Therefore, the association between lipid accumulation and mitochondrial dysfunction is not unequivocal, as indeed lipid storage and handling is energetically (ATP) expensive. Thus, our results in BAT showing increased mitochondrial function and lipid accumulation are not an exception, neither contradictory. We believe that our manuscript has now been greatly improved thanks to the editorial process and the addition of these new Figure panels asked by the reviewer and the advisor. We look forward your decision.

3rd Editorial Decision 30 March 2017

Thank you for the submission of your revised manuscript to EMBO reports. You have performed the experiments that both the referee and the advisor considered essential to complete the phenotypic characterization of the BAT-Mfn2 KO mice. You have added data showing whole-body oxygen consumption rates after  3-agonist injection as well as physical activity data. Moreover, you have rewritten and reorganized the manuscript as suggested by the referee. Given that the new data support the main conclusions of the study and that the referee is supportive of publication, we think that the manuscript is now suitable for publication in EMBO reports.

Browsing through the manuscript myself I found several issues that need to be taken care of before the manuscript can be accepted:

- Please show the p-value for Fig. S2D to unambiguously demonstrate that the difference in physical activity between wt and KO mice is not significant.

- Fig. 6J: I noticed that the increase in TG levels at 30{degree sign}C compared to 22{degree sign}C looks very similar in WT and KO mice. Even though this difference might not be statistically significant in the KO mice, you might want to mention that it follows a similar trend. Just looking at it by eye, I can hardly see a difference in serum TG levels between WT and KO.

- Please provide scale bars for the zoomed image in Fig. S3D and for Fig S3G and Fig S3G zoom

- The box in Fig. S3G does not correlate with the content of the magnified image. The latter is larger.

- Please reformat the Supplementary material as follows: You can provide up to 5 images as Expanded View. For these please follow the nomenclature Figure EV1, Figure EV2 etc. The figure legend for EV figures should be included in the main manuscript document file in a section called Expanded View Figure Legends after the main Figure Legends. Additional Supplementary material should be supplied as a single pdf labeled Appendix. The Appendix includes a table of content on the first page, all figures and their legends. Please follow the nomenclature Appendix Figure Sx throughout the text and also label the figures according to this nomenclature. For more details please refer to our guide to authors. Of course you can also supply all supplementary material in the Appendix. The advantage of the EV figures is that they are easy to access in the html version of the manuscript. Since you moved most of the data on male mice to the Supplement this would allow a fast and easy comparison between the data on females shown in the main figure to the male data in the EV figure.

I am looking forward to seeing a final version of the manuscript when it is ready.

3rd Revision - authors' response 03 April 2017

Thanks a lot for looking at our manuscript so carefully. Please find a revised version and responses to your specific points.

- Please show the p-value for Fig. S2D to unambiguously demonstrate that the difference in physical activity between wt and KO mice is not significant.

*We have added the p value for Fig S2D (now Appendix Figure S1D), using a Student t test, unpaired, wt vs KO. p=0.17.*

- Fig. 6J: I noticed that the increase in TG levels at 30{degree sign}C compared to 22{degree sign}C looks very similar in WT and KO mice. Even though this difference might not be statistically significant in the KO mice, you might want to mention that it follows a similar trend. Just looking at it by eye, I can hardly see a difference in serum TG levels between WT and KO.

*While we wanted to state that there are no differences between WT and KO by writing "without significant changes in the steady state levels of circulating TG", we agree that this sentence is not accurate enough to illustrate the mentioned trends. To clarify the sentence and comment on the fact that thermoneutrality increases TG levels in both genotypes, we wrote:*

*"Indeed, these increases were associated with a reduction in circulating cholesterol levels in BAT-MFN2-KO obese females (Figure 6I). However, no significant changes in the thermoneutrality-induced steady state levels of circulating TG were detected between WT and BAT-Mfn2-KO obese females (Figure 6J)."*

*In addition, we realized that the # on the KO bar was lost during the figure formatting. We double checked the calculations and confirmed that the induction of TG by thermoneutrality in the KO is indeed significant. This has been corrected. We thank you again for such a careful revision of the figure.*

- Please provide scale bars for the zoomed image in Fig. S3D and for Fig S3G and Fig S3G zoom

*We have provided scale bars for the zoomed image in Figure S3D and in Figure S3G.*

- The box in Fig. S3G does not correlate with the content of the magnified image. The latter is larger.

*We have erased the zoomed image in Figure S3G, as with the electron microscopy images added during the revision process, there is no need to show mitochondrial morphology by zooming immunohistochemistry images.*

- Please reformat the Supplementary material as follows: You can provide up to 5 images as Expanded View. For these please follow the nomenclature Figure EV1, Figure EV2 etc. The figure legend for EV figures should be included in the main manuscript document file in a section called Expanded View Figure Legends after the main Figure Legends.

*We will provide 2 Figures for expanded view: Previous Figure S1 is now Figure EV1 and Previous Figure S5 is now Figure EV2. Their legends are included after the main figure legends and we will upload EV figures as TIFF. Additional Supplementary material should be supplied as a single pdf labeled Appendix. The Appendix includes a table of content on the first page, all figures and their legends. Please follow the nomenclature Appendix Figure Sx throughout the text and also label the figures according to this nomenclature. For more details please refer to our guide to authors.*
*The rest of supplementary Figures are now in the appendix pdf file, including their legends.*
*Of course you can also supply all supplementary material in the Appendix. The advantage of the EV figures is that they are easy to access in the html version of the manuscript. Since you moved most of the data on male mice to the Supplement this would allow a fast and easy comparison between the data on females shown in the main figure to the male data in the EV figure. Figure S5 including the male data is now Figure EV2*

4th Editorial Decision 10 April 2017

I am very pleased to accept your manuscript for publication in the next available issue of EMBO reports. Thank you for your contribution to our journal.

EMBO PRESS

YOU MUST COMPLETE ALL CELLS WITH A PINK BACKGROUND ➜

PLEASE NOTE THAT THIS CHECKLIST WILL BE PUBLISHED ALONGSIDE YOUR PAPER


Corresponding Author Name: Orian Shirihai, Marc Liesa

Journal Submitted to: EMBO

Manuscript Number: EMBOJ-2016-94913R1

## Reporting Checklist For Life Sciences Articles (Rev. July 2015)

This checklist is used to ensure good reporting standards and to improve the reproducibility of published results. These guidelines are consistent with the Principles and Guidelines for Reporting Preclinical Research issued by the NIH in 2014. Please follow the journal's authorship guidelines in preparing your manuscript.

### A- Figures

#### 1. Data

**The data shown in figures should satisfy the following conditions:**

- the data were obtained and processed according to the field's best practice and are presented to reflect the results of the experiments in an accurate and unbiased manner.
- figure panels include only data points, measurements or observations that can be compared to each other in a scientifically meaningful way.
- graphs include clearly labeled error bars for independent experiments and sample sizes. Unless justified, error bars should not be shown for technical replicates.
- if n< 5, the individual data points from each experiment should be plotted and any statistical test employed should be justified
- Source Data should be included to report the data underlying graphs. Please follow the guidelines set out in the author ship guidelines on Data Presentation.

#### 2. Captions

**Each figure caption should contain the following information, for each panel where they are relevant:**

- a specification of the experimental system investigated (eg cell line, species name).
- the assay(s) and method(s) used to carry out the reported observations and measurements
- an explicit mention of the biological and chemical entity(ies) that are being measured.
- an explicit mention of the biological and chemical entity(ies) that are altered/varied/perturbed in a controlled manner.

- the exact sample size (n) for each experimental group/condition, given as a number, not a range;
- a description of the sample collection allowing the reader to understand whether the samples represent technical or biological replicates (including how many animals, litters, cultures, etc.).
- a statement of how many times the experiment shown was independently replicated in the laboratory.
- definitions of statistical methods and measures:
  - common tests, such as t-test (please specify whether paired vs. unpaired), simple χ2 tests, Wilcoxon and Mann-Whitney tests, can be unambiguously identified by name only, but more complex techniques should be described in the methods section;
  - are tests one-sided or two-sided?
  - are there adjustments for multiple comparisons?
  - exact statistical test results, e.g., P values = x but not P values < x;
  - definition of 'center values' as median or average;
  - definition of error bars as s.d. or s.e.m.

Any descriptions too long for the figure legend should be included in the methods section and/or with the source data.

**Please ensure that the answers to the following questions are reported in the manuscript itself. We encourage you to include a specific subsection in the methods section for statistics, reagents, animal models and human subjects.**

**In the pink boxes below, provide the page number(s) of the manuscript draft or figure legend(s) where the information can be located. Every question should be answered. If the question is not relevant to your research, please write NA (non applicable).**

### USEFUL LINKS FOR COMPLETING THIS FORM

| | |
|---|---|
| http://www.antibodypedia.com | Antibodypedia |
| http://1degreebio.org | 1DegreeBio |
| http://www.equator-network.org/reporting-guidelines/improving-bioscience-research-repo | ARRIVE Guidelines |
| http://grants.nih.gov/grants/olaw/olaw.htm | NIH Guidelines in animal use |
| http://www.mrc.ac.uk/Ourresearch/Ethicsresearchguidance/Useofanimals/index.htm | MRC Guidelines on animal use |
| http://ClinicalTrials.gov | Clinical Trial registration |
| http://www.consort-statement.org | CONSORT Flow Diagram |
| http://www.consort-statement.org/checklists/view/32-consort/66-title | CONSORT Check List |
| http://www.equator-network.org/reporting-guidelines/reporting-recommendations-for-tun | REMARK Reporting Guidelines (marker prognostic studies) |
| http://datadryad.org | Dryad |
| http://figshare.com | Figshare |
| http://www.ncbi.nlm.nih.gov/gap | dbGAP |
| http://www.ebi.ac.uk/ega | EGA |
| http://biomodels.net/ | Biomodels Database |
| http://biomodels.net/miriam/ | MIRIAM Guidelines |
| http://jjj.biochem.sun.ac.za | JWS Online |
| http://oba.od.nih.gov/biosecurity/biosecurity_documents.html | Biosecurity Documents from NIH |
| http://www.selectagents.gov/ | List of Select Agents |

### B- Statistics and general methods

| | Please fill out these boxes ➜ (Do not worry if you cannot see all your text once you press return) |
|---|---|
| 1.a. How was the sample size chosen to ensure adequate power to detect a pre-specified effect size? | The sample size was chosen according to our previous experience with this technique and other references performing similar studies (n=4-15 for in vivo studies). Per IACUC complicance, we used the minimal sample size that would give statistical significant differences for the parameters measured. |
| 1.b. For animal studies, include a statement about sample size estimate even if no statistical methods were used. | Sample sizes for each animal study has been included in the figure legends |
| 2. Describe inclusion/exclusion criteria if samples or animals were excluded from the analysis. Were the criteria pre-established? | Exclusion criteria include presence, after veterinary assesment, of infections, ulcerative dermatitis, bladder enlargements or other common alterations in this mouse strain, not specific to the genetic manipulation. Criteria was pre-established to exclude samples from mice that had methodological/technical failures of their measurements, established by the presence of controls. |
| 3. Were any steps taken to minimize the effects of subjective bias when allocating animals/samples to treatment (e.g. randomization procedure)? If yes, please describe. | While we needed the genotypes to compare littermates and therefore experimental groups were created by indentifying the mice, some in vivo measurements were done in the absence of the genotype information at the time of data collection. In addition, some experiments and measurements were performed by investigators that did not have the genotype information. |
| For animal studies, include a statement about randomization even if no randomization was used. | No randomization was used, as we needed to compare littermates of the same age and gender in each experiment. |
| 4.a. Were any steps taken to minimize the effects of subjective bias during group allocation or/and when assessing results (e.g. blinding of the investigator)? If yes please describe. | Yes, during some in vivo tests. |
| 4.b. For animal studies, include a statement about blinding even if no blinding was done | Blinding was performed in vivo and in vitro tests, including Metabolic chamber, NMR, pcr, rt-pcr, mitochondrial respiration, elisa, TG & cholesterol measurements, and cold exposure. The personnel participating/performing these assays did not have the genotypes. Other assays were not blinded. |
| 5. For every figure, are statistical tests justified as appropriate? | Yes, we used Student-t- test and ANOVA, as the data obtained through the methods used in this study have been widely established to follow a normal distribution. |
| Do the data meet the assumptions of the tests (e.g., normal distribution)? Describe any methods used to assess it. | Yes, we believe that the data meets the assumptions of the tests, per our prior experience and the references in the literature using the same methods in the same mouse strains. |
| Is there an estimate of variation within each group of data? | Yes, as standard error of the mean and/or standard deviation. |
| Is the variance similar between the groups that are being statistically compared? | It depends on the experiments, but the Student t tests in this study are performed considering that the samples have unequal variance. |

### C- Reagents

| | |
|---|---|
| 6. To show that antibodies were profiled for use in the system under study (assay and species), provide a citation, catalog number and/or clone number, supplementary information or reference to an antibody validation profile. e.g., Antibodypedia (see link list at top right), 1DegreeBio (see link list at top right). | Mfn2 (Ms, ab56889, Abcam), UCP-1 (Rb, ab10983, Abcam), Tomm20 (Rb, ab78547, Abcam), OXPHOS cocktail (Ms, Ms604/H4664, Abcam), PGAM1 (Rb, 12098S, Cell Signaling), PKM2 (Rb, 3106S, Cell Signaling), GAPDH (Rb, 2118S, Cell Signaling), HSP90 (Rb, 4877S, Cell Signaling), Porin [illegible] |
| 7. Identify the source of cell lines and report if they were recently authenticated (e.g., by STR profiling) and tested for mycoplasma contamination. | No cell lines were used in this study |

* for all hyperlinks, please see the table at the top right of the document

### D- Animal Models

| | |
|---|---|
| 8. Report species, strain, gender, age of animals and genetic modification status where applicable. Please detail housing and husbandry conditions and the source of animals. | Ucp1-cre(+/-) mice were provided by Dr. Aprahamian's lab in C57Bl6/J background and currently in Jackson laboratories (Jax Stock No 024670). Mfn2loxP mice were provided by Dr. David Chan and generated in mixed C57Bl6J/129 background (Chen et al., 2007). Ucp1-cre transgenic and Mfn2flox/flox mice were crossed to generate Ucp1-cre+/- Mfn2flox/flox (BAT-Mfn2-KO) and Ucp1-cre-/- Mfn2flox/flox (control) mice. Males and females of ages ranging 3-14 months old were used and the age is specified in each figure legend. Mice were kept under a 12:12-h dark-light period |
| 9. For experiments involving live vertebrates, include a statement of compliance with ethical regulations and identify the committee(s) approving the experiments. | All experiments were approved by the Institutional Animal Care and Use Committee (IACUC), protocol # 14855 at Boston University. |
| 10. We recommend consulting the ARRIVE guidelines (see link list at top right) (PLoS Biol. 8(6), e1000412, 2010) to ensure that other relevant aspects of animal studies are adequately reported. See author guidelines, under 'Reporting Guidelines'. See also: NIH (see link list at top right) and MRC (see link list at top right) recommendations. Please confirm compliance. | We confirm compliance to the best of our knowledge |

### E- Human Subjects

| | |
|---|---|
| 11. Identify the committee(s) approving the study protocol. | NA |
| 12. Include a statement confirming that informed consent was obtained from all subjects and that the experiments conformed to the principles set out in the WMA Declaration of Helsinki and the Department of Health and Human Services Belmont Report. | NA |
| 13. For publication of patient photos, include a statement confirming that consent to publish was obtained. | NA |

| | |
|---|---|
| 14. Report any restrictions on the availability (and/or on the use) of human data or samples. | NA |
| 15. Report the clinical trial registration number (at ClinicalTrials.gov or equivalent), where applicable. | NA |
| 16. For phase II and III randomized controlled trials, please refer to the CONSORT flow diagram (see link list at top right) and submit the CONSORT checklist (see link list at top right) with your submission. See author guidelines, under 'Reporting Guidelines'. Please confirm you have submitted this list. | NA |
| 17. For tumor marker prognostic studies, we recommend that you follow the REMARK reporting guidelines (see link list at top right). See author guidelines, under 'Reporting Guidelines'. Please confirm you have followed these guidelines. | NA |

### F- Data Accessibility

| | |
|---|---|
| 18. Provide accession codes for deposited data. See author guidelines, under 'Data Deposition'.<br><br>Data deposition in a public repository is mandatory for:<br>a. Protein, DNA and RNA sequences<br>b. Macromolecular structures<br>c. Crystallographic data for small molecules<br>d. Functional genomics data<br>e. Proteomics and molecular interactions | NA |
| 19. Deposition is strongly recommended for any datasets that are central and integral to the study; please consider the journal's data policy. If no structured public repository exists for a given data type, we encourage the provision of datasets in the manuscript as a Supplementary Document (see author guidelines under 'Expanded View' or in unstructured repositories such as Dryad (see link list at top right) or Figshare (see link list at top right). | NA |
| 20. Access to human clinical and genomic datasets should be provided with as few restrictions as possible while respecting ethical obligations to the patients and relevant medical and legal issues. If practically possible and compatible with the individual consent agreement used in the study, such data should be deposited in one of the major public access-controlled repositories such as dbGAP (see link list at top right) or EGA (see link list at top right). | NA |
| 21. As far as possible, primary and referenced data should be formally cited in a Data Availability section. Please state whether you have included this section.<br><br>Examples:<br>**Primary Data**<br>Wetmore KM, Deutschbauer AM, Price MN, Arkin AP (2012). Comparison of gene expression and mutant fitness in Shewanella oneidensis MR-1. Gene Expression Omnibus GSE39462<br>**Referenced Data**<br>Huang J, Brown AF, Lei M (2012). Crystal structure of the TRBD domain of TERT and the CR4/5 of TR. Protein Data Bank 4O26<br>AP-MS analysis of human histone deacetylase interactions in CEM-T cells (2013). PRIDE PXD000208 | NA |
| 22. Computational models that are central and integral to a study should be shared without restrictions and provided in a machine-readable form. The relevant accession numbers or links should be provided. When possible, standardized format (SBML, CellML) should be used instead of scripts (e.g. MATLAB). Authors are strongly encouraged to follow the MIRIAM guidelines (see link list at top right) and deposit their model in a public database such as Biomodels (see link list at top right) or JWS Online (see link list at top right). If computer source code is provided with the paper, it should be deposited in a public repository or included in supplementary information. | NA |

### G- Dual use research of concern

| | |
|---|---|
| 23. Could your study fall under dual use research restrictions? Please check biosecurity documents (see link list at top right) and list of select agents and toxins (APHIS/CDC) (see link list at top right). According to our biosecurity guidelines, provide a statement only if it could. | We do not anticipate that our study will fall under dual use research restrictions |
